# 3D small-scale dosimetry and tumor control of ^225^Ac radiopharmaceuticals for prostate cancer

**DOI:** 10.1038/s41598-024-70417-3

**Published:** 2024-08-27

**Authors:** Robin Peter, Anil P. Bidkar, Kondapa Naidu Bobba, Luann Zerefa, Chandrashekhar Dasari, Niranjan Meher, Anju Wadhwa, Adam Oskowitz, Bin Liu, Brian W. Miller, Kai Vetter, Robert R. Flavell, Youngho Seo

**Affiliations:** 1 Department of Nuclear Engineering, University of California, Berkeley, CA USA; 2 Department of Radiology and Biomedical Imaging, University of California, San Francisco, CA USA; 3 Department of Surgery, Cardiovascular Research Institute, University of California, San Francisco, CA USA; 4 Department of Anesthesia, University of California, San Francisco, CA USA; 5 Departments of Radiation Oncology and Medical Imaging, University of Arizona, Tucson, AZ USA; 6 Department of Pharmaceutical Chemistry, University of California, San Francisco, CA USA

**Keywords:** Radiopharmaceutical therapy, Digital autoradiography, Alpha particle therapy, Microdosimetry/small-scale dosimetry, Preclinical research, Targeted therapies, Biomedical engineering, Imaging techniques

## Abstract

Radiopharmaceutical therapy using $\upalpha$-emitting $^{225}$Ac is an emerging treatment for patients with advanced metastatic cancers. Measurement of the spatial dose distribution in organs and tumors is needed to inform treatment dose prescription and reduce off-target toxicity, at not only organ but also sub-organ scales. Digital autoradiography with $\upalpha$-sensitive detection devices can measure radioactivity distributions at 20–40 $\upmu {\hbox {m}}$ resolution, but anatomical characterization is typically limited to 2D. We collected digital autoradiographs across whole tissues to generate 3D dose volumes and used them to evaluate the simultaneous tumor control and regional kidney dosimetry of a novel therapeutic radiopharmaceutical for prostate cancer, [^225^Ac]Ac-Macropa-PEG_4_-YS5, in mice. 22Rv1 xenograft-bearing mice treated with 18.5 kBq of [^225^Ac]Ac-Macropa-PEG_4_-YS5 were sacrificed at 24 h and 168 h post-injection for quantitative $\upalpha$-particle digital autoradiography and hematoxylin and eosin staining. Gamma-ray spectroscopy of biodistribution data was used to determine temporal dynamics and $^{213}$Bi redistribution. Tumor control probability and sub-kidney dosimetry were assessed. Heterogeneous $^{225}$Ac spatial distribution was observed in both tumors and kidneys. Tumor control was maintained despite heterogeneity if cold spots coincided with necrotic regions. $^{225}$Ac dose-rate was highest in the cortex and renal vasculature. Extrapolation of tumor control suggested that kidney absorbed dose could be reduced by 41% while maintaining 90% TCP. The 3D dosimetry methods described allow for whole tumor and organ dose measurements following $^{225}$Ac radiopharmaceutical therapy, which correlate to tumor control and toxicity outcomes.

## Introduction

Radiopharmaceutical therapy (RPT) is a cancer treatment modality that utilizes a molecular targeting strategy to deliver a therapeutic radioisotopic emission to tumor cells. Both $\upalpha$- and $\upbeta$-particle modes have demonstrated therapeutic effects in clinical trials for a range of oncologic diseases, including metastatic castration-resistant prostate cancer (mCRPC), an aggressive disease with a 2–3 year survival rate and 50% 6-month mortality^1,2^. $\upbeta$-emitting [^177^Lu]Lu-PSMA-617 is already in use for mCRPC patient care, but 20–40% of treated patients do not respond to it^3^. $\upalpha$-particle RPT ($\upalpha$RPT) is an alternative with shorter range ($<{100}\,\,\upmu {\hbox {m}}$) and higher linear energy transfer that may overcome improve therapeutic outcomes even in cases of $\upbeta$-particle radioresistance^4,5^.

The word *dose* in this manuscript refers to the radiation absorbed dose (J/kg or Gy), which in radiation-based therapies is the key metric that links the intensity of administered treatment to tumor- and organ-effects. Despite evidence of dose-effect relationships for a variety of radiopharmaceuticals (RPs), de-/escalation strategies based on quantitative dosimetry and accepted biological effect metrics such as tumor control probability (TCP) are still scarce^6^. For $\upalpha$RPT, dose-effect dosimetry is confounded by the often heterogeneous distribution of absorbed dose in tumors and tissues resulting from the short range of $\upalpha$-particles and nonuniform expression of molecular targets^7,8^. These effects occur at scales smaller than the resolution of clinical PET and SPECT ($<{100}\,\,\upmu {\hbox {m}}$). Additionally, for $\upalpha$-emitters with long-lived radioactive progeny such as ^225^Ac (progeny $^{213}$Bi , $t_{1/2} = {45.6}\,{\hbox {min}}$), the release of free radionuclides through nuclear recoil poses a toxicity risk independent of the targeting efficacy^9^.

Digital autoradiography (DAR) is a common ex vivo tool to assess activity distributions and dosimetry of preclinical samples and patient biopsies at the microscale (20–40 $\upmu {\hbox {m}}$)^10,11^. Compared with in vivo preclinical PET or SPECT imaging, DAR offers higher spatial resolution and sensitivity (up to 50% geometric efficiency) due to the contact imaging geometry and high efficiency for alpha particle detection. The ability to directly image the therapeutic alpha-particle also simplifies quantitative dosimetry and avoids inaccuracies from the use of imaging surrogates^12^. Autoradiography is therefore the primary tool to study intratumoral and sub-organ dose heterogeneity in preclinical $\upalpha$RPT studies at therapeutic injected activities ($<{37}$ kBq).

Digital autoradiographs (DARs) have been correlated with histological stains (typically with hematoxylin and eosin, or H&E), gamma-ray spectroscopy, and even high-resolution MRI data to interrogate sub-organ and sub-tumor effects^13,14^, typically using one or a few representative slices due to labor or instrument field-of-view constraints. Observable features are thus limited to a single plane per slice due to the 2D nature of the modality. The potential benefit to full 3D investigation of $\upalpha$-radiopharmaceutical agents ($\upalpha$RPs) in tissues and tumors has not been assessed, nor is the method straightforward. It is theoretically possible to collect enough consecutive DARs from a tissue to assemble a 3D absorbed dose rate distribution^15^, but the procedure is practically prohibitive: imaging a typical 5-mm diameter tissue at ${10}\,\,\upmu {\hbox {m}}$ slice thickness would require 500 cryotome sections. Sparse sampling results in loss of the 3D activity information necessary to calculate absorbed dose. We propose that if adjacent slices are assumed to be similar, as is common in analysis of 2D DARs, 3D quantitative digital autoradiography is feasible with sampling.

Here, we combine quantitative methods for DAR dosimetry, gamma-ray biodistribution, and H&E stain analysis with our proposed 3D DAR method to study [^225^Ac]Ac-Macropa-PEG_4_-YS5 in murine tumors and kidneys and relate the 3D sub-organ absorbed dose distribution to predicted biological outcome. YS5 is a human monoclonal antibody that we identified previously that binds to a tumor-selective epitope of CD46^16^. Our group has developed several novel $\upalpha$RPs utilizing YS5, including [^225^Ac]Ac-Macropa-PEG_4_-YS5, which has shown specific uptake in prostate cancer models and demonstrates promise for ^134^Ce/$^{225}$Ac theranostic development with ^134^Ce as the PET diagnostic and $^{225}$Ac as the therapeutic agent^17,18^.

This work comprises 3D volumetric DAR dosimetry for spatial information, gamma-ray spectroscopy for temporal information, and co-registration and segmentation of H&E images for morphological information. It is a start-to-finish methodological framework for the assessment of small-scale effects (sub-organ dosimetry and voxel-based TCP) when studying the efficacy and toxicity of $\upalpha$RPs .

## Methods

### Ethics approval

The animal experiments were approved by and carried out in compliance with the Institutional Animal Care and Use Committee (IACUC) and established guidelines at the Laboratory Animal Resource Center (LARC), University of California, San Francisco, CA. The study design and methods follow recommendations in the ARRIVE guidelines.

### Experimental design

Immunocompromised Nu/nu mice (5–6 weeks old, Strain: 002019, Jackson Laboratories) were used for subcutaneous xenografts. Each mouse was subcutaneously inoculated with 2.5 million 22Rv1 cells mixed with Matrigel (Corning, #354230) in a 1:1 ratio. Tumor growth was monitored for 21 days until the tumors reached a volume of 0.4–0.6 cc. All animals for prostate cancer models used in our studies were male mice.

22Rv1 xenograft-bearing mice received an intravenous injection of 18.5 kBq of [^225^Ac]Ac-Macropa-PEG_4_-YS5 via the tail vein and were sacrificed at two time points: 24 h post-injection (p.i.) and 168 h (7 d) post-injection. $^{225}$Ac was in equilibrium at the time of injection. Euthanization was performed with a high dose of isoflurane (5% for 10 min), followed by cervical dislocation. Blood, tumors, kidneys, and other selected organs were collected for biodistribution (BioD: NaI automatic gamma counter, Hidex), and only tumors and kidneys were subjected for autoradiography (iQID: ionizing-radiation quantum imaging detector, QScint Imaging Solutions, LLC). Consecutive tissue slices were stained with hematoxylin and eosin (H&E) using a standard protocol. Antibody conjugation, $^{225}$Ac radiolabeling, and [^225^Ac]Ac-Macropa-PEG_4_-YS5 synthesis followed the procedure described previously^17^.

In total, four cohorts of identically prepared mice are described in this study: animals for DAR method comparison ($N=4$), animals for 3D DAR ($N=4$), animals for BioD-based $^{213}$Bi corrections ($N=8$), and animals in a 7-d BioD study to determine the time-dose-rate curves for [^225^Ac]Ac-Macropa-PEG_4_-YS5 in the mice ($N=17$).

### iQID digital autoradiography imaging

An ionizing-radiation quantum imaging detector (iQID) camera DAR device (QScint Imaging Solutions, LLC) was used to obtain high-resolution (voxel size $39\,\,\upmu {\hbox {m}}\times 39\,\,\upmu {\hbox {m}}\times 210\,\,\upmu {\hbox {m}}$) images of the instantaneous spatial distribution of $\upalpha$-particle emissions in tissues at the start of the acquisition. The iQID camera can be used at 10–40 $\upmu {\hbox {m}}$ effective voxel size. In this study, a larger stage (80 mm diameter) was used to increase the number of tissue samples that could be measured simultaneously, with the trade-off of increasing the effective voxel size.

After sacrifice, tissue samples were prepared in an Optimum Cutting Temperature (OCT) medium, sliced using a cryotome to 10 $\upmu {\hbox {m}}$ thickness, and mounted on the iQID camera for imaging. The iQID camera uses a disposable $\upalpha$-sensitive scintillator (ZnS:Ag film EJ-440; Eljen Technology) and light-amplifying optical components to image scintillation light onto a 2448 $\times$ 2048 px CMOS camera with a CMOSIS CMV4000 CMOS sensor (Grasshopper© 3, FLIR Integrated Imaging). Activity images are obtained from single-particle event maps using the ROI segmentation and registration procedures as published in our open-source Python toolkit^19^, then decay-corrected to the time of sacrifice. An additional correction factor for the device frame-rate (1.09) was derived for $^{225}$Ac due to the rapid decay of progeny ^217^At ($t_{1/2} = {32}\,{\hbox {ms}}$), which occurs on the order of the iQID frame rate (25 FPS = 40 ms/frame) (see Supplement).

As in previous work with ^211^At^19^, $10^7$ $\upalpha$-particle primaries were generated in Monte Carlo framework GATE v9.0^20^ to simulate the decay of ^225^Ac. Alpha-particle emissions from the ^225^Ac decay chain were simulated in a 181-$\upmu {\hbox {m}}$ cube of 1-$\upmu {\hbox {m}}$ water voxels using the emstandard_opt3 physics list and 10 nm range cuts. The energies and branching ratios of the alpha-particle primaries were provided by the Lund/LBNL Nuclear Data Search (^225^Ac: 25%, ^221^Fr: 25%, ^217^At: 25%, ^213^Bi: 0.52%, ^213^Po: 24.48%)^21^. Progeny were assumed to be in secular equilibrium, since iQID measurements were taken long enough after sacrifice for free $^{213}$Bi to decay significantly (> 5 h). Only the alpha particles were generated as primaries. The kernel was averaged radially and binned to the voxel size of the iQID image stack (XY: 39 $\upmu$m; Z: 10 $\upmu$m).

#### iQID calibration

The quantitative accuracy of ^225^Ac measurements using iQID was calibrated using droplet samples of known radioactivity. Solutions of 185 Bq/$\upmu$L were prepared and serially diluted by factors of two down to 5.78 Bq/$\upmu$L with small volumes reserved at each dilution. 2-$\upmu$L droplets of each concentration were prepared ($N=3$ per concentration), counted in a Hidex NaI(Tl) automatic gamma counter (60 s, 175–250 keV and 385–490 keV windows with Gaussian and linear background fits), pipetted onto ZnS:Ag scintillator paper, and evaporated in a fume hood at room temperature, leaving circular samples of 370, 185, 92.5, 46.25, 23.13, and 11.56 Bq as calculated from the stock dilution. $^{225}$Ac was provided as a dissolved chloride salt in water, and therefore it does not vaporize at room temperature during the procedure. The swatch was measured in iQID at 25 FPS for 24 h. For activities below 46.25 Bq, the mean spatial pileup loss was $23.8\% \pm 0.7\%$, yielding an absolute efficiency of 38% when including 50% geometric efficiency. Although greater saturation occurred at higher activities, these are beyond the range of tissue measurements in this study. The complete calibration results are shown in Supplementary Fig. S4.

### 2D to 3D DAR

DAR-based $\upalpha$RP dosimetry conventionally requires a series of around 10 consecutive slices to be imaged per dose-rate measurement^15,22^. The co-registered activity volume is used for dose-point kernel (DPK) convolution or Monte Carlo (MC) simulation. If one assumes that neighboring slices are nearly identical since the slice thickness is small (10 $\upmu {\hbox {m}}$), absorbed dose-rate can be estimated with only one slice by digitally duplicating the measured slice to generate the DPK convolution input volume^19^. To validate the technique (“cloning method”) for [^225^Ac]Ac-Macropa-PEG_4_-YS5 in a mouse model, ten consecutive slices ($10 \times {10}{\hbox{ }\upmu\hbox {m}} = {100}{\hbox{ }\upmu\hbox {m}}$ total) from each mouse kidney and tumor were cut, imaged, and digitally re-registered ($N=4$ mice). The dose rate of the central slice was compared between the sequential and cloning methods.

To assess 3D dose volumes, mice were identically prepared ($N=4$), but instead single tissue slices were extracted at 200 $\upmu {\hbox {m}}$ intervals from kidneys and tumors to yield 3D volumes of 20–30 slices per tissue (voxel size $39\,\upmu {\hbox {m}}\times 39\,\upmu {\hbox {m}}\times 210\,\upmu {\hbox {m}}$). Spatial dose rates were estimated in each slice using the cloning method and DPK convolution. This procedure will be referred to as 3D digital autoradiography (3D DAR) and produces 3D digital autoradiographs (3D DARs). At 200 $\upmu {\hbox {m}}$ sampling rate in a 5-mm diameter tissue, the cloning method reduces the slices that must be prepared for 3D DAR from 250 to 25. 3D DAR figures were rendered with 3D Slicer, an open-source image analysis software package^23^.

### Tumor control probability

Tumor control probability (TCP) is a statistical predictor of treatment efficacy based on whether tumor cells survive the treatment, where $\text {TCP}=1$ indicates that all malignant cells die. We use the formalism reviewed by Spoormans et al^10^, where TCP in a heterogeneous DAR is the product of voxel control probabilities (VCPs). The VCP nomenclature is discussed further in the Supplement. A voxel *i* containing $n_i$ cells is assumed to contain uniform dose $D_i$, and the surviving fraction *S* is based on the linear quadratic (LQ) probability model:

1 $$\begin{aligned} S = e^{-\alpha D - \beta D^2} \approx e^{-\alpha D}\nonumber \\ \text {VCP}(D_i) = e^{-n_i S(D_i)}\nonumber \\ \text {TCP} = \prod _i \text {VCP}(D_i). \end{aligned}$$

The above simplification is reasonable for $^{225}$Ac RPs, which mainly deliver dose through high-LET $\upalpha$-particle emissions for which the radiosensitivity parameter $\alpha>> \beta$. We used $\alpha = {1.8} \,\,{\hbox {Gy}}^{-1}$, based on an in vitro survival assay with [^225^Ac]Ac-Macropa-PEG_4_-YS5  (Supplementary Fig. S1)^17^.

### $^{225}$Ac decay chain

Redistribution of free $^{213}$Bi was measured with time-sensitive gamma-counting ($^{213}$Bi correction cohort: $N=4$ at each of 24 h and 168 h p.i.), similar to Seoane et al^24^. We assumed secular equilibrium between $^{225}$Ac ($t_{1/2} = {9.9} {\hbox { d}}$) and ^221^Fr ($t_{1/2} = {4.8} {\hbox { min}}$), but not between $^{225}$Ac and ^213^Bi ($t_{1/2} = {45.6}\,{\hbox {min}}$), since measurements occurred $>30$ min after sacrifice. Derivations and some nuances are discussed in the Supplement.

A measurement at time *t* post-sacrifice provided $A_a(t)$ and $A_b(t)$, the respective $^{225}$Ac and $^{213}$Bi activities, which were related to $A_a(0)$ and $A_b(0)$ at the instant of sacrifice ($t=0$) by:

2 $$\begin{aligned} \frac{A_b(t)}{A_a(t)} = \biggr (\frac{A_b(0)}{A_a(0)} - \frac{\lambda _b}{\lambda _b - \lambda _a}\biggr ) e^{-(\lambda _b-\lambda _a)t} + \frac{\lambda _b}{\lambda _b - \lambda _a} \end{aligned}$$

All measurements corresponded to animals sacrificed at the same time post-injection. Equation (2) is equivalent to

3 $$\begin{aligned} A_b(0) = A_b(t)e^{\lambda _b t} - \frac{\lambda _b}{\lambda _b - \lambda _a} A_a(t) \left( e^{\lambda _b t} - e^{\lambda _a t}\right) . \end{aligned}$$

$A_b(t)/A_a(t) = 1$ is the condition that describes secular equilibrium between $^{225}$Ac and ^213^Bi at any time *t*. For tissues with $A_b(t)/A_a(t) > 1$ (Fig. S2), the difference between the total activities at sacrifice, $A_b(0)-A_a(0)$, was the quantity of free $^{213}$Bi present at that moment (e.g. due to redistribution from other tissues).

### Gamma-ray spectroscopy

Gamma-ray emissions from organs and tumors were counted in a Hidex NaI(Tl) automatic gamma counter between 0.5 and 3 h post-sacrifice, allowing 60 s active counting time per tissue. Net counts were recorded in energy windows corresponding to ^221^Fr (168–268 keV) and ^213^Bi (370–510 keV), using a least-squares Gaussian distribution with linear background to correct for ambient background (^213^Bi) and down-scatter (^221^Fr) in each energy window (Supplementary Fig. S2). Counts were corrected by their respective branching ratios, decay times, and energy-dependent detector efficiencies, determined by a known-activity detector calibration using the same procedures.

### Macro-to-micro dosimetry

The temporal evolution of activity was estimated using macroscopic gamma-counting measurements and a macro-to-micro approach^25^. The absorbed dose value in each DAR voxel was extrapolated by scaling the dose-rate curve measured within whole tumors and kidneys by a factor *c* based on the mean dose-rate of the DAR measurement at one time-point (24 h or 168 h p.i.), assuming that the activity does not significantly redistribute over time. The dose-rate curve was modelled with time-dependent BioD from 1 d, 2 d, 4 d, and 7 d p.i. ($N=17$).

Energy from the $^{225}$Ac decay chain was assumed to deposit entirely within the tumor (which showed $A_b(t)/A_a(t) = 1$ within uncertainties), but in kidneys, we separated $^{225}$Ac contributions from free $^{213}$Bi and its products. Resulting dose-rates were fit to bi-exponential curves using least-squares optimization. We extracted extrapolation factors $c_{24h}$ and $c_{168h}$,

4 $$\begin{aligned} c_{24h} = D/\dot{D}_{24h}, \qquad c_{168h} = D/\dot{D}_{168h}, \end{aligned}$$

where *D* was the total integrated dose under the time-rate curve (TRC) to six half-lives, and $\dot{D}_{24h}$ (for example) was the dose-rate measured at 24 h p.i by BioD. iQID dose-rate DARs from 24 h p.i. were scaled by $c_{24h}$ to obtain the voxel distribution of total absorbed dose $D_i$, and similarly for 168 h p.i. DARs by $c_{168h}$.

### Histological staining

Tissue slices consecutive with each sample series (sequential-method validation mice) or with each tissue slice (3D DAR mice) were stained with hematoxylin and eosin (H&E) using a standard protocol. Images were acquired with Octopus-Versa Slide Scanner (Leica).

### Image segmentation and registration

#### Tumors

Cell nuclei in tumor H&E images were segmented using a custom ImageJ-Fiji macro based on watershed segmentation and the Analyze Particles function (Supplementary Fig. S3). These cell nuclei maps and iQID images were initially co-registered using automated rigid-body transformations with mean-squared-error intensity comparison, as described previously^19^, but more precise registration was needed to match $n_i$ cells in a voxel to dose $D_i$ to calculate TCP. After the initial rigid-body registration, the two images were manually aligned with affine transformations using Bigwarp^26^, a landmark-based deformation tool in ImageJ-Fiji^27^. To minimize interpolation errors, the DAR was treated as the reference image where possible. When DARs were transformed, the sum of pixel values was preserved using a scaling factor according to the difference before and after transformation. External edges of the tissue were preferred as landmarks to avoid biasing co-registration of internal structures receiving dose.

Tissue slices from both modalities (iQID and H&E) sometimes contained damaged or folded sections from the cryosectioning procedure. Identifiable damage was masked out of the TCP calculations, but the difference in total tissue extent sometimes hindered the registration. If a visibly adequate co-registration could not be achieved, the slice was omitted from analysis. To reduce the sensitivity of the calculation to registration error, we applied a 5 px $\times$ 5 px erosion mask to the edge of the contour outlining $n_i > 0$ pixels.

#### Kidneys

Kidney H&E images were manually segmented into four regions: cortex; the combined inner and outer stripes of medulla (ISOM/OSOM); the combined inner medulla and papilla (IM/Pa); and the combined vasculature and renal pelvis (V/Pe), using the reference histology images provided by NIH’s National Toxicology Program^28^. We reduced the uncertainty in segmentation by combining the outer and inner stripes of medulla into one segment, and defined the boundary with the cortex as the presence or absence of glomerules. Similarly, we did not distinguish where the inner medulla and papilla ended or began and masked them as one segment. Automated rigid-body transformations were sufficient for approximate alignment of the sub-organ regions when registering anatomical masks with 3D DARs.

We evaluated our DAR-based sub-organ dosimetry results from 24 h p.i. next to a regional S-value dosimetry model developed by Vargas et al.^14^. This model takes the total activity as measured by BioD and distributes it according to the relative activity ratios in each compartment. We calculated this ratio using DARs from across the whole kidney volume and applied the published S-value calculations according to the procedure described by the authors. Since we did not separate the ISOM and OSOM, or the IM from the papilla, these segments were combined and their S-values averaged. Vasculature was assigned to the compartment within which it was found for this analysis, since it was not segmented in the reference.

### Statistical analysis

Results reported as $x \pm \sigma$ describe the mean value *x* and one standard deviation $\sigma$. Results of the form $x~(x_1, x_2)$ show asymmetric uncertainties, where $x_1$ is the lower bound and $x_2$ is the upper bound propagated from dose calculations. For sub-organ dosimetry using H&E stains, damage to the kidneys during cryotome slicing resulted in only $N=1$ mouse per time point, for which *x* and $\sigma$ are calculated using contralateral samples (left and right kidneys).

## Results

All mentions of *dose* refer to absorbed dose (Gy), with no radiation weighting or relative biological effectiveness factor.

### 2D to 3D DAR

**Fig. 1:**
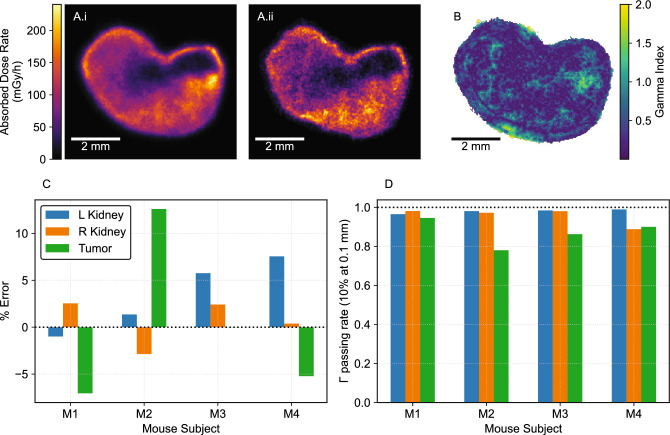
Cloning method characterization. (**A**) Example dose-rate images (24 h p.i. tumor) calculated using the sequential (**i**) and cloning (**ii**) methods. (**B**) Gamma index analysis (local normalization, 0.1 mm, 10%). (**C**) Percentage error of the mean voxel dose-rate of cloning method compared to sequential method images from DAR mouse studies at 1 d (subjects M1, M2) and 7 d (M3, M4) post-injection. (**D**) Gamma index passing rates.

Figure 1A-B shows an example comparison between the absorbed-dose-rate DAR calculated using the sequential and cloning methods for a 24 h p.i. tumour. Across subjects (kidneys and tumors at both time points), the cloning method calculated the mean dose-rate of the sequential method with an accuracy of ${4.1\% \pm 3.7}{\%}$ (Fig. 1C). Gross features were captured, but the approximation was noisier and over- or under-emphasized high-activity regions. We assessed the spatial accuracy with $\gamma$ analysis, a difference- and distance-based metric for similarity between two dose distributions that is used to evaluate clinical external-beam radiation therapy plans^29,30^. ${97\%\pm 3}{\%}$ of dose-rate pixels in kidneys were accurately calculated ($\gamma < 1$), using a tolerance of 10% within three pixels (117 $\upmu {\hbox {m}}$) and local normalization (Fig. 1D). Higher discrepancy was observed in tumors ($\gamma < 1:{87\%\pm 6}{\%}$), which reflects the fact that heterogeneities between slices are not preserved when using the cloning method approximation.

### $^{213}$Bi spectroscopy

**Figure 2:**
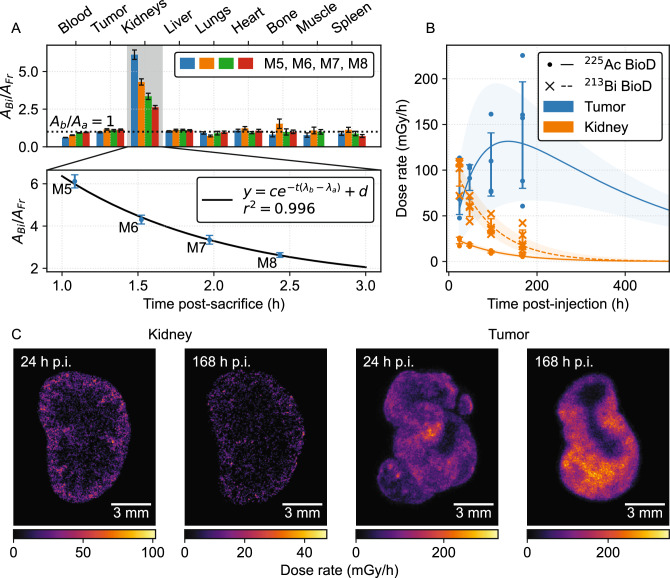
Temporal studies of [^225^Ac]Ac-Macropa-PEG_4_-YS5 in 22Rv1 xenograft-bearing mice. (**A**) $^{213}$Bi correction example using gamma-ray spectroscopy for 24 h p.i. mice (subjects M5-M8). Activity ratios of the two $^{225}$Ac daughters demonstrates clearance of free $^{213}$Bi in the blood through the kidneys ($N=4$). The decay of the $A_b(t)/A_a(t)$ ratio following sacrifice follows Eq. (2) despite variable uptake between subjects. (**B**) Time-dose-rate curves (18.5 kBq, $N=17$), with contributions from free $^{213}$Bi and $^{225}$Ac in kidneys separated. (**C**) Representative DARs from kidneys and tumors at two time points.

BioD results from 24 h p.i. are shown in Fig. 2A ($N=4$). The ratio $A_b(t)/A_a(t)$ was compared to unity at $t={1} {\hbox {h}}$ post-sacrifice (first bar in each 4-bar set) to determine deviation from secular equilibrium in tissues. In kidneys, $A_b/A_a = {6.1 \pm 0.3} > 1$ indicated substantial free redistributed ^213^Bi. Blood measurements were deficient in $^{213}$Bi ($A_b/A_a = {0.62 \pm 0.01} < 1$), which suggests that $^{213}$Bi was cleared from blood through the kidneys. Tumors were in secular equilibrium (${0.97 \pm 0.04}$). At 168 h p.i., $A_b/A_a$ for tumor, kidney, and blood was ${1.06 \pm 0.04}$, ${5.30 \pm 0.56}$, and ${1.03 \pm 0.10}$, respectively. Both early and late time points showed tumors in secular equilibrium between $^{225}$Ac and ^213^Bi, while kidneys collected redistributed ^213^Bi. Lowered levels of $^{213}$Bi in the blood at the early time point had equilibrated by the late time point.

The decay of $A_b(t)/A_a(t)$ in kidneys post-sacrifice followed Eq. (2) with a goodness-of-fit coefficient of determination $r^2 = {0.996}$ ($N=4$ mice, 60 s active counting per tissue). The decreasing signal in the kidneys was used to measure the free ^213^Bi using Eqs. (2) and (3). $A_b(0)/A_a(0)$ was calculated from Eqs. (2) and (3) for 24 h p.i. mice as ${14.7 \pm 0.2}$ and ${14.0 \pm 1.2}$, respectively, which agree within $1\sigma$. Agreement was also observed at 168 h p.i. (${17.1 \pm 1.8}$ and ${15.6 \pm 2.4}$). In both cases, the more precise result was used for subsequent analyses. Greater uncertainties for the experimental fit at 168 h p.i. are attributable to the lower overall activities remaining in the system and a poorer exponential fit due to an oversight in the 168 h p.i. data that resulted in a narrower time window for the measurements (only 8 min between mice).

### Single-time-point dosimetry

Figure 2B shows time-dose-rate curves (TRCs) using the approach illustrated in Fig. 2A to separate kidney absorbed dose contributions into $^{225}$Ac and free $^{213}$Bi components. No $^{213}$Bi data from 24 h p.i. were available from this cohort due to a 7-h delay in measurement. The 24 h p.i. data shown were extrapolated from the measured $^{225}$Ac activity $A_a$ and the calculated correction factor $A_b(0)/A_a(0)$.

The dominant uncertainty in the TRCs was the variable uptake between animal subjects, shown as $1\sigma$ error bars around the mean absorbed dose rate. Individual subject data points are shown instead of the mean dose rates themselves. We calculated the total absorbed dose for each TRC as the integrated area, with uncertainties as the dose from upper- and lower-bound curves defined by modulating the fitting parameters by $\pm 1\sigma$. Table 1 summarizes the calculated doses and conversion factors *c* for each TRC with bound-based uncertainties.

**Table 1:** Absorbed dose calculations and correction factors from integration of BioD dose-rate curves (Fig. 2B).

Tissue	Radioisotope	Dose (mGy/kBq)	$c_{24h} = D/\dot{D}_{24h}$ (mGy/mGy-h)	$c_{168h} = D/\dot{D}_{168h}$ (mGy/mGy-h)
Tumor	$^{225}$Ac	3540 (2120, 4840)	870 (520, 1190)	474 (283, 647)
Kidneys	$^{225}$Ac	142 (121, 168)	114 (97, 135)	406 (345, 480)
Kidneys	$^{213}$Bi	466 (349, 627)	84 (63, 113)	361 (271, 486)

Use of the extrapolation factor assumes that the intra-organ and intra-tumor spatial activity do not change over time. Figure 2C shows representative DARs for kidneys and tumors at 24 h and 168 h post-injection. The ratio between mean absorbed dose-rates in each renal compartment (cortex, ISOM/OSOM, IM/papilla, and V/Pe) was (1, 0.50, 0.88, 1.19) at 24 h p.i., and (1, 0.60, 0.83, 1.06) at 168 h p.i., indicating that similar compartmental distribution was preserved. In tumors, both time-points exhibited morphology-dependent activity distribution with low dose in the necrotic core, but higher tumor saturation was observed at 168 h post-injection.

**Table 2:** Absorbed dose comparisons between modalities and tissues using single-point dosimetry.

Tissue	Modality	$^{225}$Ac Dose (Gy/kBq)	$^{213}$Bi Dose (Gy/kBq)*	Total Dose (Gy/kBq)
Kidneys	DAR ($N=2$)	0.09 (0.07, 0.11)	0.26 (0.18, 0.37)	0.35 (0.25, 0.48)
Kidneys	BioD ($N=8$)	0.08 ± 0.02	0.32 ± 0.14	0.40±0.14
Tumor	DAR ($N=3$)	2.8 ± 0.2	–	2.8 ± 0.2
Tumor	BioD ($N=8$)	3.1 ± 1.0	–	3.1 ± 1.0

*Dose due to $\upalpha$-particles from redistributed free $^{213}$Bi and its associated progeny $^{213}$Po. For DAR, separation of $^{213}$Bi (and $^{213}$Po) dose is based on $A_b(0)/A_a(0)$ ratio calculated in the ^213^Bi spectroscopy section. Significant redistribution was not observed to tumors (Fig. 2A)

### 3D kidney dosimetry

The mean kidney absorbed dose from 18.5 kBq [^225^Ac]Ac-Macropa-PEG_4_-YS5 was 6.4 (4.6, 8.9) Gy and 7.5 ± 2.2 Gy from DAR and BioD, respectively (Table 2). 75% (73%, 78%) (DAR) and 78 ± 7 % (BioD) of the total mean dose was due to the decay of free ^213^Bi, where the $^{213}$Bi correction to iQID DARs was provided by the $A_b(0)/A_a(0)$ ratio. The spatial distribution of free $^{213}$Bi was not obtained from these DARs, which were imaged several days post-sacrifice. The reported mean values of the two modalities differed by 15% and agree within $1\sigma$ of BioD statistical uncertainties.

**Fig. 3:**
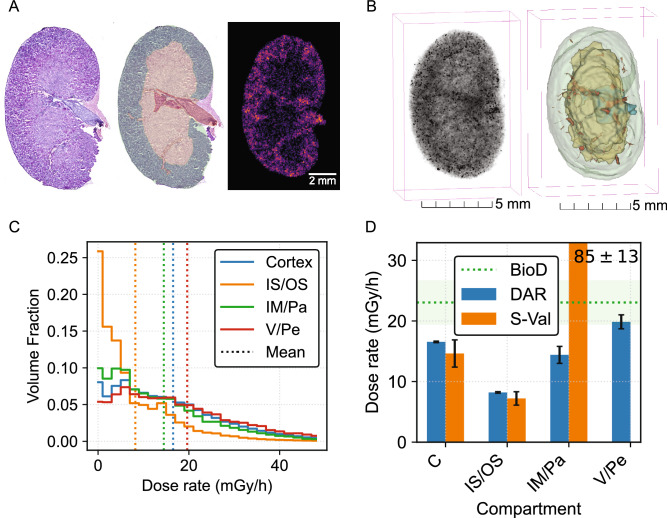
3D kidney dosimetry and segmentation. (**A**) Example sub-organ dosimetry slice from a 24 h p.i. kidney, showing H&E image, anatomical mask, and 2D iQID DAR. (**B**) 3D DAR and H&E-based anatomical mask for the whole kidney, comprising 23 slices. (**C**) Dose-rate volume histogram (DrVH) due to $^{225}$Ac for segmented regions of both kidneys at 24 h p.i. (**D**) Comparison between iQID DAR, regional S-values^14^, and BioD for kidney dosimetry.

Figure 3A illustrates an example H&E stained slice, the anatomically segmented regions (cortex, ISOM/OSOM, IM/papilla, and V/Pe), and the corresponding iQID DAR. All 23 slices from a 24 h p.i. kidney were combined to create the 3D DAR and co-registered 3D anatomical model in Fig. 3B.

Dose-rate volume histograms (DrVHs) from each anatomical compartment are shown in Fig. 3C. Average $^{225}$Ac dose-rate in the renal cortex doubled that in the medulla at 24 h p.i. (16.6 ± 0.1 mGy/h vs. 8.2 ± 0.1 mGy/h) and was 67% higher at 168 h p.i. (4.24 ± 0.04 mGy/h vs. 2.53 ± 0.07 mGy/h). The inner medulla and papilla mean dose-rate was similar to that in the cortex (13%, 18% less for respective time-points). Blood vessels and the renal pelvis collected comparatively high amounts of $^{225}$Ac at the two time points (20 ± 1 mGy/h, 4 ± 1 mGy/h). These results show high $^{225}$Ac concentrations at key transport locations: blood vessels, cortex, and the renal pelvis. Intact antibody-based radiopharmaceutical compounds such as [^225^Ac]Ac-Macropa-PEG_4_-YS5 may be too large for filtration and thus stagnate in the glomeruli or remain in the blood.

The iQID DAR and regional S-value methods agreed within uncertainties for cortex and ISOM/OSOM regions (Fig. 3D). However, the S-value calculation indicated an IM/Pa dose rate exceeding 5 times that of the DAR method (bar extends beyond figure limits). The BioD mean dose rate (23.1 ± 3.6 mGy/h), which assumed full energy deposition of all decay products within the tissue, was naturally uniform and higher than the iQID and S-value estimates, except for the IM/Pa (Fig. 3D).

### Tumor dosimetry and TCP

**Fig. 4:**
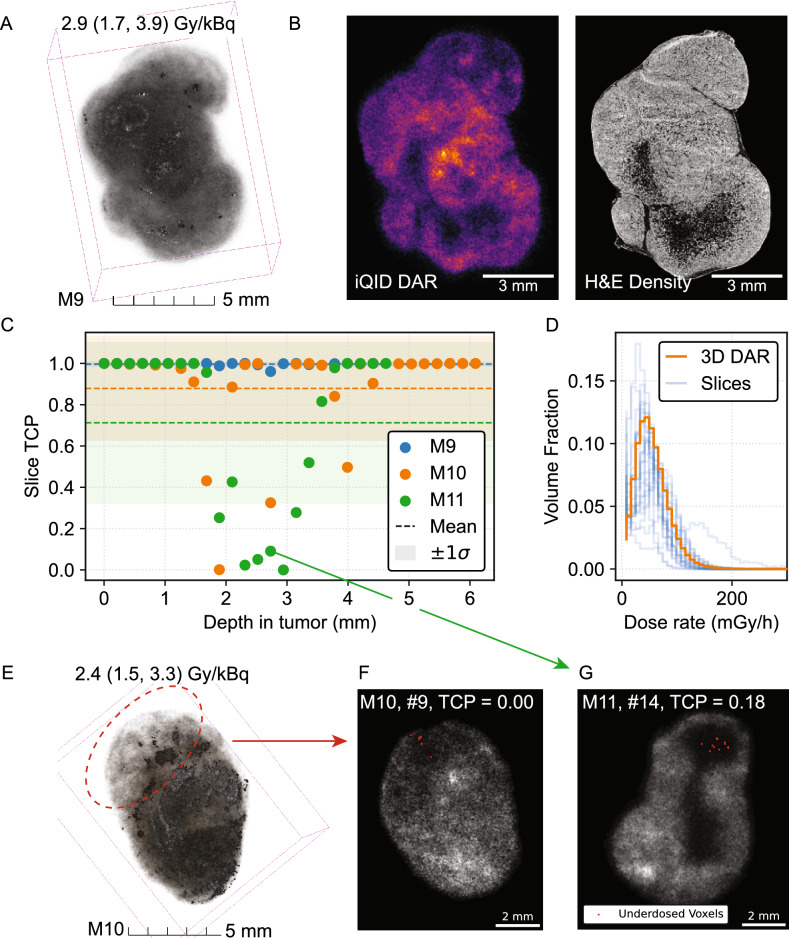
Tumor dosimetry and TCP calculation. (**A**) 3D DAR of 24 h p.i tumor (M9). (**B**) Co-registered iQID dose-rate DAR with corresponding cell nucleus density image segmented from H&E. (**C**) Slice-by-slice TCP ($N=3$ mice with 21-27 slices each). (**D**) Tumor DrVH for individual slices compared to 3D DAR volume. (**E**) 3D DAR of 24 h p.i. tumor (M11). (**F**–**G**) Illustrative low-TCP slices. Dose-rate DAR is shown in gray-scale, with underdosed voxels ($<0.95$ VCP) indicated.

Tumors received an average of 50.8 ± 4.1 Gy (DAR) and 57.4 ± 18.5 Gy (BioD) between the two modalities (2.8 ± 0.2 Gy/kBq or 3.1 ± 1.0 Gy/kBq: Table 2). Figure 4A–D summarizes tumor dosimetry and the TCP calculation process for a 24 h p.i. tumor, including the 3D DAR (Fig. 4A), example registered iQID and cell density images from H&E (Fig. 4B), TCP values for individual slices (Fig. 4C), and DrVHs for individual slices and the total volume for an example 24 h p.i. tumor (Fig. 4D). This analysis was conducted for $N=3$ mice (labelled M9, M10, and M11), including two at 24 h p.i. (TCP: ${1.00\pm 0.01}$, ${0.88\pm 0.25}$) and one at 7 d p.i. (${0.71\pm 0.39}$). Values are cited as the mean and standard deviation of TCP calculated for individual slices.

Despite heterogeneous dose distribution and cold spots, high tumor control is attained in mouse M9 because low-dose regions correspond to the necrotic core of the tissue with few cell nuclei (Fig. 4B). Between the two 24 h p.i. mice, lower mean dose (17% less) and heterogeneous uptake of the radiopharmaceutical in non-necrotic regions resulted in decreased TCP. The red dashed circle in the 3D DAR (Fig. 4E) indicates a region of reduced uptake in one lobe of the tumor. Figure 4F shows a representative gray-scale DAR with low slice-TCP (0.00), with voxels with $\text {VCP}_i < 0.95$ indicated in red. Fig. 4G shows the same for a low-TCP slice from 7 d post-injection.

**Fig. 5:**
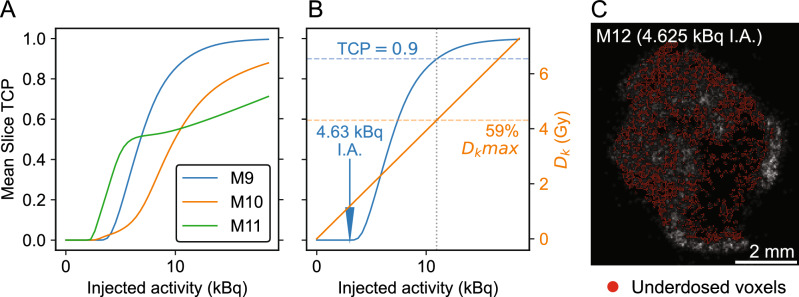
De-escalation predictive calculation. (**A**) Calculation of mean-slice TCP for a range of injected activities. (**B**) In subject M9, de-escalation calculation suggests that kidney dose may be reduced by 41% while maintaining 90% TCP. For 4.625 kBq I.A., the model predicts 0% TCP. (**C**) Slice from 4.625 kBq I.A. experiment, showing gray-scale DAR dose-rate with underdosed voxels ($<0.95$ VCP) indicated. The calculated TCP of 0% is consistent with the model’s prediction.

The mean slice-TCP and kidney dose were estimated for a range of injected activities (IA) from 0 to 18.5 kBq, assuming that dose scales linearly with IA and maintains the same organ and sub-organ spatial distribution (Fig. 4A). We then calculated a predictive de-escalation scheme using the highest TCP tumor (M9, Fig. 4B). In this simple model, a reduced IA of 10.9 kBq maintained a TCP of 0.9 with a 41% reduction in kidney dose. 75% IA reduction (4.625 kBq) was predicted to yield non-controlled tumors (TCP $=0$). To test this, one additional mouse was prepared and analyzed with 4.625 kBq IA. We observed sparse radiopharmaceutical uptake, in comparison to the 18.5 kBq cohort, and TCP was calculated as zero for all slices in agreement with the model (Fig. 5B–C). This calculation is consistent with a previous therapy and survival study with [^225^Ac]Ac-Macropa-PEG_4_-YS5 in mice, which found that 4.625 IA treatment extended survival and inhibited tumor growth for 41 days compared to saline, followed by tumor regrowth^17^. Subjects M10 and M11 did not reach 0.9 TCP and were not evaluated for de-escalation.

## Discussion

Microdosimetry and small-scale dosimetry are crucial to understanding the biological effect and treatment strategy of $\upalpha$-particle radiopharmaceuticals by linking spatial absorbed dose distribution to tumor kill or organ toxicity^8,31,32^. In this work, we have demonstrated how three staples of pre-clinical RP studies—gamma-ray biodistribution, immunohistological stains, and digital autoradiography—may be combined to assess tumor control probability and produce DrVHs of anatomical compartments. To our knowledge, this is the first study to generate and analyze 3D-DAR in entire organs and tumors, and to calculate voxel-based TCP for experimental $^{225}$Ac $\upalpha$RP measurements simultaneously with sub-organ kidney dosimetry. Since tumor dose from radiotherapeutics is limited by the tolerance of normal tissues, it is necessary and natural that small-scale tumor and organ dosimetry should be evaluated concurrently.

The study is mainly limited by the fact that no direct-comparison survival-and-treatment study of [^225^Ac]Ac-Macropa-PEG_4_-YS5 was conducted at the 18.5 kBq IA level, and therefore we cannot draw final conclusions about the treatment outcome and toxicity. However, results from 4.625 kBq treatments are available and can provide some insight to our results^17^. The 4.625 kBq [^225^Ac]Ac-Macropa-PEG_4_-YS5 treatment extended survival and inhibited tumor growth for 41 days compared to saline, but tumors ultimately regrew. This is consistent with our simple predictive model and the added 4.625 kBq mouse assessed with the DAR-TCP method. For sub-organ dosimetry, we found that [^225^Ac]Ac-Macropa-PEG_4_-YS5 was predominantly in the cortex, vasculature, and pelvis-adjacent structures. This agrees with the survival study, which observed mild to moderate renal toxicity and histopathological reduction and atrophy of the cortical glomerules and tubules. The simple de-escalation scheme was calculated for only one mouse with high TCP ($>0.9$), since de-escalation is not a goal for sub-controlled tumors. Therefore, we can only treat this method as a proof of concept. With the DAR-TCP and sub-organ methodological framework established, future treatment studies can both utilize and validate the method by calculating TCP in cohorts treated at several injected activity levels and identifying potential correlation to disease progression and mortality. Exploration of these methods is increasingly relevant given the positive outcomes reported by clinical de-escalation trials^33^.

We found several important nuances when using VCP-product TCP as a metric for $\upalpha$RP treatment efficacy. In this framework, if every 10-$\upmu {\hbox {m}}$ slice has a high $\text {TCP}=0.95$, where $\text {TCP}=1$ is complete tumor control, then the cumulative 3D TCP of a 5-mm tumor would be $(0.95)^{50} = 0.08$. Given the tumor inhibition observed in treatment studies, we suspect that the mean TCP per slice may better reflect the treatment efficacy^17^. The TCP metric is conservative, formulated based on the probability of 100% cell kill, and thus does not necessarily capture meaningful outcomes such as prolonged survival, as we found with our comparison to the 4.625 kBq study. Additionally, we used conservative assumptions to estimate the radiosensitivity parameter $\alpha$ (see Supplement), so the actual biological impact and tumor control are likely to be higher than predicted when correcting for binding saturation, sterilized cells, well geometry, and dose delivered by recoiling nuclei. We have not considered hypoxia, variable radiosensitivity, or repopulation and repair^10^.

An inherent drawback of ex vivo DAR and BioD studies is that the animal must be sacrificed to conduct a measurement, precluding monitoring of the same animal over time and demanding costly increases in sample size to minimize inter-subject variability. Moreover, the treatment cannot be adjusted on a subject-specific basis unless tissue biopsies are obtained. In this study, we utilized the minimum number of mice feasible to obtain a representative data set, resulting in large uncertainties attributable to single-point pharmacokinetics and the resulting inter-subject variability (Fig. 2B). These challenges are characteristic of the macro-to-micro dosimetry approach. Although the animal cohort was small, each tissue or tumor was assessed over 20-30 independent slices to evaluate intra-subject variability and the benefit of a 3D DAR approach. Fig. 4C and the 3D DAR panels in the Supplement demonstrate that a single 2D slice might not include structural or dose-distribution features present elsewhere in the 3D volume.

An in vivo study of the $\upalpha$RP characterizing the continuous pharmacokinetics over time within subjects, if one can be achieved given the low administered activities, could improve the precision of the macro-to-micro approach. Our group is working to develop ultra-high-sensitivity gamma-ray imaging techniques for $\upalpha$RPT^12,34–36^. In vivo PET and SPECT imaging surrogates for $^{225}$Ac RPs are also under investigation in our group and elsewhere^17,37^. An imaging surrogate is a chemically similar diagnostic radiopharmaceutical that may be injected at higher activities to obtain a tracer distribution correlated with the $\upalpha$RP kinetics at millimeter or sub-millimeter resolution (e.g., replacement of $^{225}$Ac with ^133^La or ^134^Ce/^134^La for PET, or ^226^Ac for SPECT). This spatial resolution may be sufficient to observe gross sub-organ features such as renal cortex-medulla separation, with the advantage of monitoring the same subject over time. The utility of surrogate imaging techniques is limited by the chemical similarity of the surrogate to the therapeutic isotope, the complication of dosimetry (requiring studies to correlate imaging biokinetics to therapeutic absorbed dose), and the lack of information provided by the surrogate about the distribution of recoil progeny. These imaging strategies are complementary to the methods described here, and can be used for in vivo imaging and ultimately for applications in clinical dosimetry^31,38^.

This work complements the study by Mellhammar et al, who used a MC-simulation DAR-TCP approach with ^177^Lu-PSMA-617 extrapolated to [^225^Ac]Ac-PSMA-617^13^. We demonstrated experimentally that, as the authors simulated, low tumor penetration reduces TCP for the same IA level. Our approach differed in that quantitative single-particle DARs of the $\upalpha$-emitting RP were measured, instead of using a relative-intensity $\upbeta$-particle DAR as a proxy for the spatial distribution of $\upalpha$-particles. We also used 3D DAR for TCP (improving statistical count and morphological assessment), included concurrent sub-organ kidney dosimetry, and accounted for both biological and physical clearance with an experimental TAC curve for the macro-to-micro conversion.

These voxel-TCP approaches may help estimate the biological outcome of doses in de-escalation studies. Although tumor dose is heterogeneous in Fig. 4B, the low-dose region corresponds to the necrotic core of the tissue with few cell nuclei, resulting in high tumor control. Conversely, de-escalation reduces tumor penetration and increases RP sparsity, affecting tumor management, as shown in Fig. 5C. DAR-based studies can explore these effects during drug development. Although we demonstrated the concept for de-escalation analysis in Fig. 5, a rigorous de-escalation model would require more refined uncertainty analysis and a larger cohort, as described above.

For ^225^Ac RPT, off-target toxicity rather than cytotoxic efficacy may be the current challenge limiting routine clinical use. The high tumor control observed here was accompanied by 350–400 mGy/kBq (6.4–7.5 Gy) absorbed dose to kidneys. Gamma-ray spectroscopy suggested that this was largely attributable to redistributed ^213^Bi. Inclusion of a biological weighting factor such as relative biological effectiveness (RBE) or radiation weighting ($w_R$) representing the double-strand breaks from $\upalpha$-particles could bring the 6.4–7.5 Gy $\upalpha$-particle dose above the 15 Gy threshold associated with nepropathy in external-beam radiation therapy^39^. Since our spectroscopy approach only predicts the total activity correction and not the sub-organ spatial distribution of free ^213^Bi, early-time DAR or other separation techniques^40^ might reveal different critical sub-structures than those we identified.

We characterized the cloning method performance to evaluate the assumption in DAR dosimetry that adjacent slices are functionally identical. The method is accurate within 10% for mean dose-rates, but differences between slices do affect spatial dose distribution in tumors (${87\%\pm 6}{\%}$ $\gamma$-passing rate). Still, the approximation procedure allowed the rapid collection of 3D DARs, which revealed spatial variation in [^225^Ac]Ac-Macropa-PEG_4_-YS5 dose within tissues. The identical-slices assumption thus appears suitable, and perhaps necessary, to generate 3D DARs for sub-organ anatomical dosimetry, but stochastic cellular microdosimetry would require a high-resolution, multi-slice source volume for accurate results. We speculate that this method only holds because of the “small-scale” (not truly microdosimetric) nature of the analysis. If a higher-resolution iQID setting or device were used, such that individual kidney tubules or glomeruli were identifiable, then the assumption that adjacent slices are replicates of each other is unlikely to be true.

## Conclusion

We developed a method for 3D digital autoradiography (3D DAR) and combined it with advanced gamma-ray spectroscopy and histological segmentation to conduct small-scale dosimetry in murine studies of $\upalpha$-emitting [^225^Ac]Ac-Macropa-PEG_4_-YS5. Tumor control and 3D sub-organ kidney absorbed dose distributions were evaluated at the voxel level (39 $\upmu {\hbox {m}} \times 39 \, \upmu {\hbox {m}} \times 210 \,\upmu {\hbox {m}}$). These methods provide an important framework to assess treatment outcomes and organ risk for $^{225}$Ac radiopharmaceutical studies.

### Supplementary Information

Supplementary Information.

## Data Availability

All analyzed data (digital autoradiograph images, H&E binary images, biodistribution data) needed to evaluate the conclusions in the paper are present in the main text or the Supplementary Materials. Unprocessed data (raw digital autoradiography list-mode data, full-resolution color H&E images, and raw biodistribution spectra) are archived locally on long-term data storage and are available upon request. Some data may be subject to materials transfer agreements (MTA) between institutions or individual organizations. All code is available upon request and/or is being maintained at https://github.com/robin-peter/iqid-alphas.
