# Supplementary material for: 3D small-scale dosimetry and tumor control of 225Ac radiopharmaceuticals for prostate cancer
Source: Sci Rep. 2024 Aug 27;14:19938. doi: 10.1038/s41598-024-70417-3 (PMC11358493; doi:10.1038/s41598-024-70417-3)
Supplement: Supplementary file 1 — Supplementary Information. [file 41598_2024_70417_MOESM1_ESM.pdf]

July 2, 2024

Supplementary Materials for **3D small-scale dosimetry and tumor control of ^{225}Ac radiopharmaceuticals for prostate cancer**

Robin Peter[1,2,†,*], Anil P. Bidkar[2,†], Kondapa Naidu Bobba[2], Luann Zerefa[2], Chandrashekhar Dasari[3], Niranjan Meher[2], Anju Wadhwa[2], Adam Oskowitz[3], Bin Liu[4], Brian W. Miller[5], Kai Vetter[1], Robert R. Flavell[2,6,*], and Youngho Seo[1,2,*]

[†] *These authors contributed equally to this work.*

[1] *Department of Nuclear Engineering, University of California, Berkeley, CA, USA*

[2] *Department of Radiology and Biomedical Imaging, University of California, San Francisco, CA, USA*

[3] *Department of Surgery, Cardiovascular Research Institute, University of California, San Francisco, CA, USA*

[4] *Department of Anesthesia, University of California, San Francisco, CA, USA*

[5] *Departments of Radiation Oncology and Medical Imaging, University of Arizona, Tucson, AZ, USA*

[6] *Department of Pharmaceutical Chemistry, University of California, San Francisco, CA, USA*

[*] *Corresponding authors:* `rpeter@berkeley.edu`, `Robert.Flavell@ucsf.edu`, `Youngho.Seo@ucsf.edu`

1. Tumor control probability formalism

In a uniform-dose distribution, for n_0 clonogenic tumor cells, the Poisson-model uniform-dose TCP is given by

$$\mathrm{TCP} = e^{-n_0 S}, \tag{1}$$

where S is the linear quadratic (LQ) probability model of survival for the cells receiving uniform dose D, as described in the main text. However, to compute a similar metric in a heterogeneous DAR, which we will call TCP*, a voxel i containing n_i cells is assumed to contain uniform dose D_i. TCP* is therefore calculated as the product of the uniform voxel control probabilities (VCPs):

$$\begin{aligned} \mathrm{VCP}(D_i) &= e^{-n_i S(D_i)} \\ \mathrm{TCP}^* &= \prod_i \mathrm{VCP}(D_i). \end{aligned} \tag{2}$$

The distinction between TCP* and TCP reflects the subtlely that the uniform-TCP value is *not* recovered in the case of a VCP-based calculation in a uniform dose distribution. An example of the numeric consequences of this fact is mentioned in the discussion. However, for convention and clarity, all mentions of TCP in the rest of the text refer to VCP-product TCP* as above.

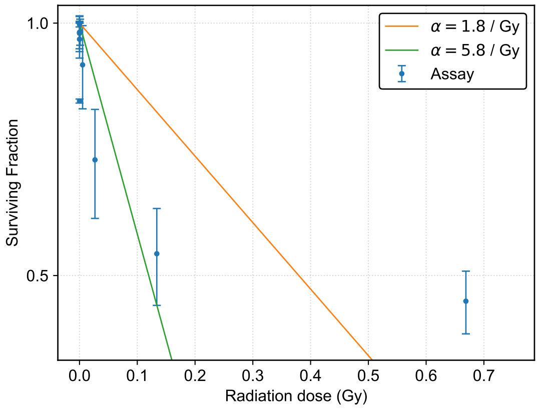

Figure S1: Converted cell survival curve of [^{225}Ac]Ac-Macropa-PEG$_4$-YS5in 22Rv1 cells based on Fig. 4B from [1].

2. Cell survival assay from [1]

A cell-killing assay, shown in Fig. 4B in [1], was used to estimate the α-radiosensitivity parameter of 22Rv1 cells to [^{225}Ac]Ac-Macropa-PEG$_4$-YS5. Relevant method details are quoted here:

> 22Rv1 cells were cultivated in 96-well plates at a density of 2,000 cells per well. The cells were treated with varying concentrations of [^{225}Ac]Ac-Macropa-PEG$_4$-YS5 (ranging from 3.7 x 10-7 to 3.7 kBq) for 96 h. After the treatment, cell viability was measured using the cell titer glo reagent (Fisher scientific, Mfr. No. Promega G7570) per the manufacturer's instructions.

The plate (Corning Inc. Costar REF 3603 96 Well Assay Plate) had a bottom growth area of 0.32 cm^2, and 100 µL of total liquid volume was applied. As a first-order approximation, cells were assumed to receive the same dose as the water, assuming full energy deposition of all alpha-particles in the ^{225}Ac decay chain over 96 h. This assumption likely underestimates the cell kill due to the actual accumulation of the labelled radiopharmaceutical on the cell membrane.

The linear quadratic model, which is well established in literature for cell survival assays, does not predict saturation at high dose, as is shown in Fig. 4B of the reference. However, there is binding saturation in a cell culture, shown in Ref. Fig. 4A. For the cell survival curve, we excluded the last data point at 50 nCi/mL, as this value falls well beyond the threshold for 90% binding saturation (22 nCi/mL, calculated from Ref. Fig. 4A). The resulting curve is shown in Fig. S1. A linear-quadratic model fit applied to the values yields $\alpha = 1.8/\text{Gy}$. A fit without the last (likely also saturated) data point yields $\alpha = 5.8/\text{Gy}$, but we used $\alpha = 1.8/\text{Gy}$ for our modelling to maintain a conservative approach (underestimating rather than overestimating tumor control). A clonogenic assay using a non-membrane permeable chelator such as EDTA/DTPA, in conjunction with a Monte Carlo simulation accounting for the geometry and alpha-particle range, would be a more accurate approach to compute the cell survival curve, but it is beyond the scope of this paper. For this study, we accept the cell kill underestimates from both the saturation and homogeneous dose assumption as a conservative approach to therapy optimization.

3. ^{225}Ac decay chain

The decay chain of ^{225}Ac is shown in Supplemental Fig. S2. At the time of animal sacrifice, biological processes halt, and the decay of ^{225}Ac and production of its daughters follow exponential decay laws. For a two-isotope decay chain, the time-dependent activity of progeny $A_b(t)$ ($t_{1/2} = \ln 2/\lambda_b$) is related to the activity of the parent $A_a(t)$ ($t_{1/2} = \ln 2/\lambda_a$) by:

$$A_b(t) = A_a(0)\frac{\lambda_b}{\lambda_b - \lambda_a}(e^{-\lambda_a t} - e^{-\lambda_b t}) + A_b(0)e^{-\lambda_b t}. \tag{3}$$

The time of sacrifice defines $t = 0$ [2].

We assume that ^{225}Ac and ^{221}Fr are in secular equilibrium, as are ^{221}Fr and ^{217}At, since the time of measurement (0.5-3 h post-sacrifice, p.s.) exceeds the six daughter half-lives needed to reach equilibrium (30 min). Therefore, the activity of ^{225}Ac is measurable as the activity of ^{221}Fr. Moreover, the activities of ^{225}Ac and ^{213}Bi are related as if direct parent and progeny, and may be defined as A_a and A_b in Eqn. 3.

A gamma-counting measurement at time t with appropriate corrections provides $A_a(t)$ and $A_b(t)$. We are interested in determining $A_a(0)$ and $A_b(0)$, the respective ^{225}Ac and ^{213}Bi activities at the instant of sacrifice. With some algebraic manipulation, Eqn. 3 may be rewritten:

$$\frac{A_b(t)}{A_a(t)} = \left(\frac{A_b(0)}{A_a(0)} - \frac{\lambda_b}{\lambda_b - \lambda_a}\right)e^{-(\lambda_b - \lambda_a)t} + \frac{\lambda_b}{\lambda_b - \lambda_a} \tag{4}$$

Equivalently,

$$A_b(0) = A_b(t)e^{\lambda_b t} - \frac{\lambda_b}{\lambda_b - \lambda_a}A_a(t)(e^{\lambda_b t} - e^{\lambda_a t}) \tag{5}$$

In both cases, $A_a(0) = A_a(t)e^{\lambda_a t}$ by simple decay correction. In a given tissue system, we assume that activity from ^{213}Bi has two components: a secular equilibrium component, due to freely decaying ^{225}Ac localized in the tissue, and a free ^{213}Bi component, due to redistribution to or from other tissues. $A_b(t)/A_a(t) = 1$ is the condition that describes secular equilibrium at any time t, including $A_b(0)/A_a(0) = 1$. Therefore, for tissues with $A_b(t)/A_a(t) > 1$ (Fig. S2), the difference between the total activities at sacrifice, $A_b(0) - A_a(0)$, is the quantity of free ^{213}Bi present at that moment.

Equation 4 is a convenient form to apply as an empirical fit to data at multiple time points; for example, in a sequential BioD measurement of organs from multiple animals sacrificed at the same time, or a sequential counting of the same animal. The equation can be written as $A_b(t)/A_a(t) = Ce^{-(\lambda_b - \lambda_a)t} + D$, where C is the fit parameter, and the desired $A_b(0)/A_a(0) = C + \lambda_b/(\lambda_b - \lambda_a)$. Note that the decay coefficient in the exponent, $\lambda_b - \lambda_a$, is not a free parameter and is constrained to the half-lives of ^{213}Bi and ^{225}Ac . If only a single measurement is available, the analytical decay correction in Eqn. 5 can be used. This analytical correction is illustrated in Supplementary Fig. S2 for initial conditions with and without free ^{213}Bi in the tissue at the time of sacrifice.

The method discussed above is similar to that in Seoane et al [3] but is expressed in different formalism that eases generalization to other parent-progeny isotopes. The ratio $A_b(t)/A_a(t)$ in Eqn. 3 is useful to check whether deviation from secular equilibrium is

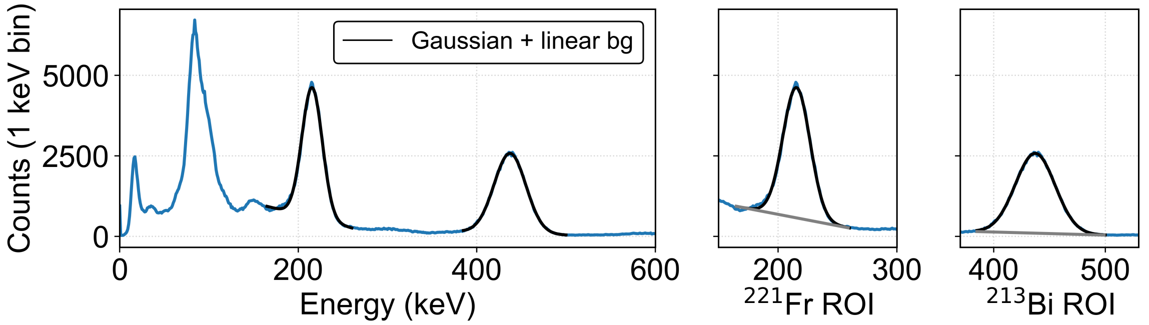

Figure S2: Illustration of counting and fitting procedure for gamma-ray spectroscopy measurements.

observed and determine which initial condition shown in Fig. S5 is relevant. Eqn. 5, with $A_a(0)$ subtracted as discussed, reduces to Seoane Eqn. 7 in the case where $\lambda_b/(\lambda_b - \lambda_a) \longrightarrow 1$ (1.003 for ^{225}Ac and ^{213}Bi). Instead of a scalar down-scatter correction in the ^{221}Fr window, a generalized Gaussian fit is applied to isolate net counts from a linear scatter "background."

4. Image segmentation and registration

4.1 Tumor cell segmentation

The custom ImageJ/FIJI macro used for segmentation of tumor H&E images is shown below.

```
// Inputs: user prompt to select A. directory to load images from, and B. directory in which to store results
// Generates one .csv file per image, with area, X coord, and Y coord (centroid) of each detected cell nucleus.
// Adapted from https://visikol.com/blog/2022/10/04/creating-imagej-macros-with-user-inputs/

// Dialogue box for user input
path=getDirectory("image directory");
Dialog.create("Select image directory and save path");
Dialog.addDirectory("Image Path", path);
Dialog.addDirectory("Save Path", path);
Dialog.show();

// Get user input values
path=Dialog.getString();
savepath=Dialog.getString();

//Initialize lists
img_list=getFileList(path);
img_list=Array.sort(img_list);

//Loop through list of images
for (i=0; i<img_list.length; i++)
{
    current_img=img_list[i];
```

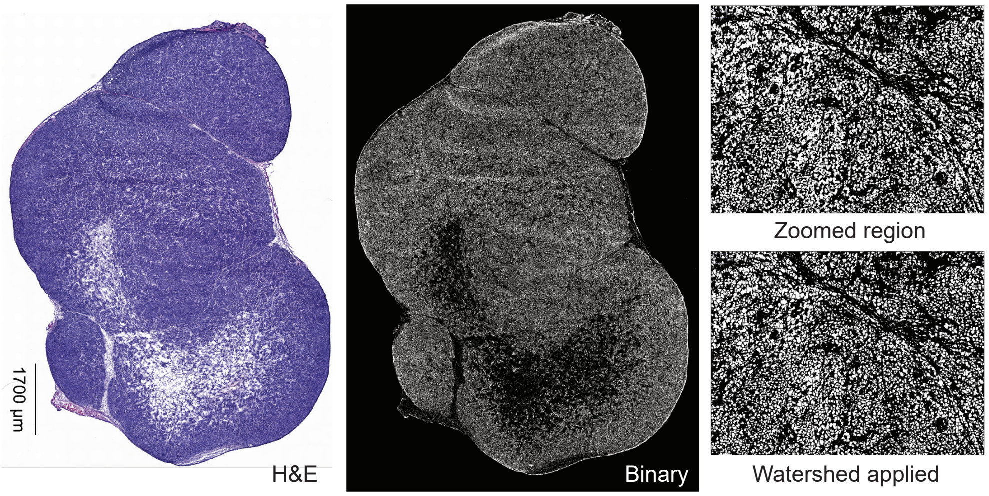

Figure S3: Illustration of tumor segmentation using ImageJ/FIJI.

```
24     img_name = File.getName(img_list[i]);
25     img_name=substring(img_name,0,lengthOf(img_name)-4);
26     print("Loading: "+path+"\\"+current_img);
27     open(path+"\\"+current_img);
28     selectImage(img_list[i]);
29     run("8-bit");
30     setAutoThreshold("Default no-reset");
31     setThreshold(0, 90, "raw");
32     setOption("BlackBackground", true);
33     run("Convert to Mask");
34     run("Watershed");
35     run("Set Measurements...", "area centroid redirect=None decimal=3");
36     run("Analyze Particles...", "size=0-500 display clear summarize");
37     selectWindow("Results");
38     saveAs("Results", savepath+"Results_"+img_name+".csv");
39     close("*");
40     close("Results");
41 }
42 selectWindow("Summary");
43 saveAs("Results", savepath+"Summary.csv");
44 print("Run finished after "+img_list.length+" images.")
```

The threshold pixel intensity value of 90 (Line 31) was manually determined by inspecting the intensity histogram and resulting binarizations of several sample images. Fig S3 shows the intermediate steps of the process.

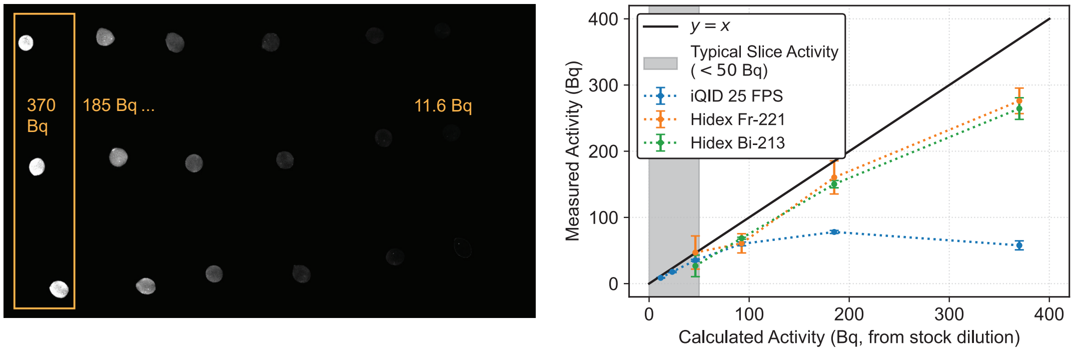

Figure S4: iQID calibration of ^{225}Ac using serial dilutions. (Left) iQID image showing relative count intensity of serial dilution droplets from 370 to 11.56 Bq. (Right) Calibration curve of iQID measured activity at 25 FPS after geometric efficiency and radioactive decay correction, compared to activity measured using Hidex NaI(Tl) gamma-counter at 218 keV (^{221}Fr) and 440 keV (^{213}Bi). Activities below 46.25 Bq were not recorded in Hidex due to sensitivity limitations.

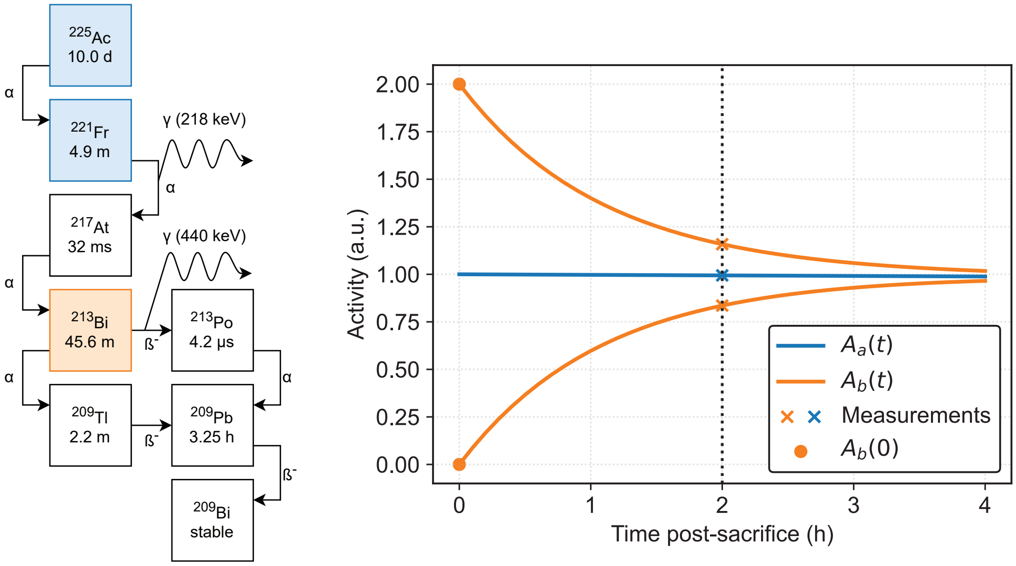

Figure S5: ^{225}**Ac decay chain and algebraic** ^{213}**Bi decay-correction.** Measured counts of the two gamma-rays from ^{221}Fr and ^{213}Bi ($\times$) are used to calculate the free ^{213}Bi activity at the time of sacrifice ($\bullet$). Activity curves for two possible initial conditions are shown, representing tissues with (above) and without (below) free ^{213}Bi present.

5. iQID frame-rate correction for ^{217}At decay

The decay from ^{221}Fr to ^{213}Bi through ^{217}At emits two α-particles, where the half-life of the $^{217}\text{At} \longrightarrow {}^{213}\text{Bi}$ decay is on the order of the iQID frame-rate at 32.3 ms (25 FPS = 40 ms frames; Supp. Fig. S5). For pixels substantially larger than the nuclear recoil range (< 100 nm), it is thus possible for both α-particles to be emitted in the same frame and same pixel. These will be recorded as only one event, effectively missing the second particle. We calculated a scalar correction based on the frequency of this occurrence, derived below.

Suppose $t_0 = 0$ is the start of the exposure window, and $t_1 = 40$ ms is the end. What is the probability that a ^{221}Fr decay at time $t \in [0, t_1]$ will be followed by a ^{217}At decay within the same window?

Since the ^{221}Fr half-life ($t_{1/2} = 4.8$ min) is long compared to the exposure window (ms), assume there is uniform probability for decay to occur at time dt within $[0, t_1]$.

Probability of radioactive decay for any t : $\quad P(t) = 1 - e^{-\lambda t}$

(A) Probability that ^{221}Fr decay happens within the window at time dt : $\dfrac{dt}{t_1}$

(B) Probability that the subsequent ^{217}At decay occurs by t_1 : $P(t_1 - t)$

The probability of a double-decay P_d in the same exposure window is the integral of (A) multiplied by (B).

$$P_d = \int_0^{t_1} P(t)\frac{dt}{t_1} = \frac{1}{t_1}\int_0^{t_1}(1 - e^{-\lambda(t_1 - t)})dt$$
$$= 1 - \frac{1}{\lambda t_1} e^{\lambda(t - t_1)}\Big|_0^{t_1} = 1 - \frac{1}{\lambda t_1}(1 - e^{-\lambda t_1})$$

For $t_1 = 40$ ms (25 fps), $P_d = 0.329$.

For ^{225}Ac measurement, the probability of an α-particle event being attributable to ^{221}Fr is one in four, $P_f = 0.25$. This is renormalized according to P_d:

$$sP_f^* = P_f/(P_f + 0.25 + 0.25(1 - P_d) + 0.25) = 0.272.$$

Therefore, in 27.2% of measured events, P_d = 32.9% of these have a missing associated ^{217}At decay. The scalar correction factor is $1 + P_d P_f^* = 1.09$.

6. Extended Data

Here we show the iQID activity DARs prior to quantitative corrections, alignment, registration, DPK, etc. 24 h p.i. kidneys from Mouse 2 were too damaged by cryotoming to use.

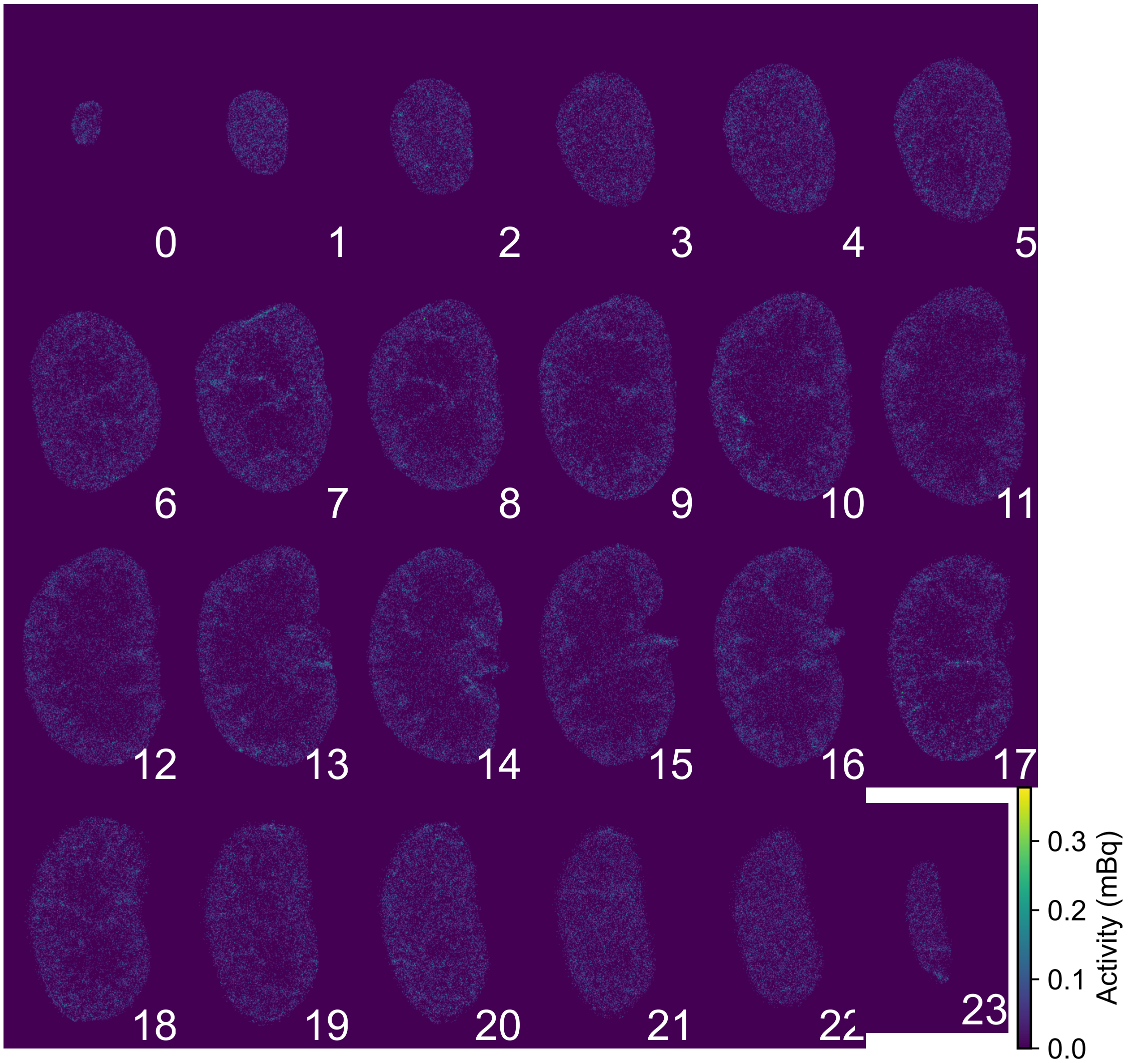

Figure S6: 24 h p.i. left kidney iQID DARs for Mouse 1 of 1.

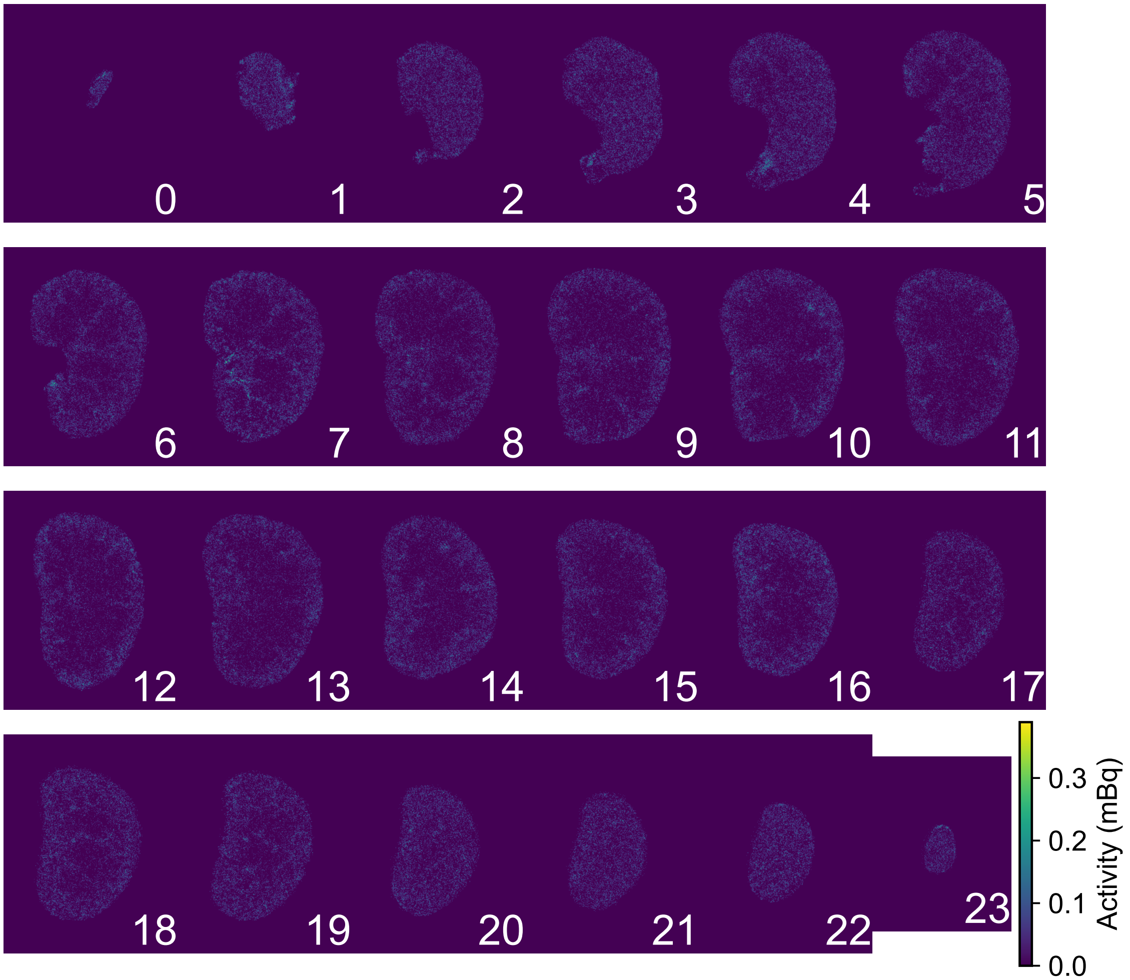

Figure S7: 24 h p.i. right kidney iQID activity DARs for Mouse 1 of 1.

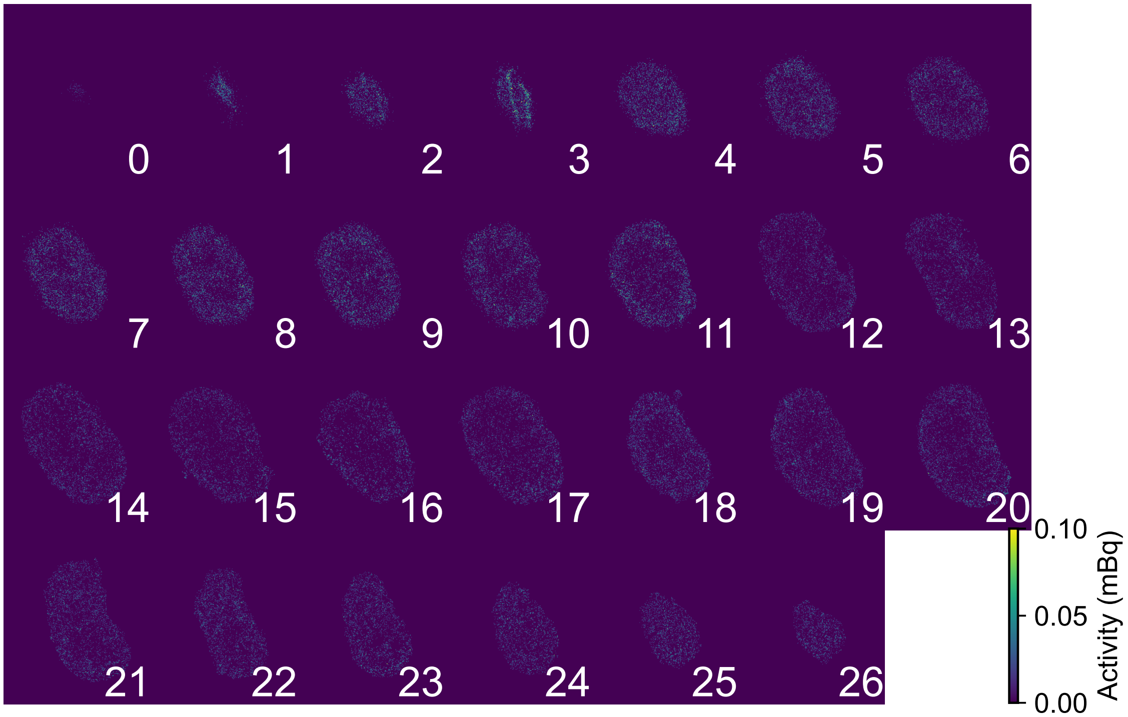

Figure S8: 168 h p.i. left kidney iQID activity DARs for Mouse 1 of 1. Streaking in slice 3 indicates crumpling of the tissue, so a duplicate of Slice 2 was used for analysis.

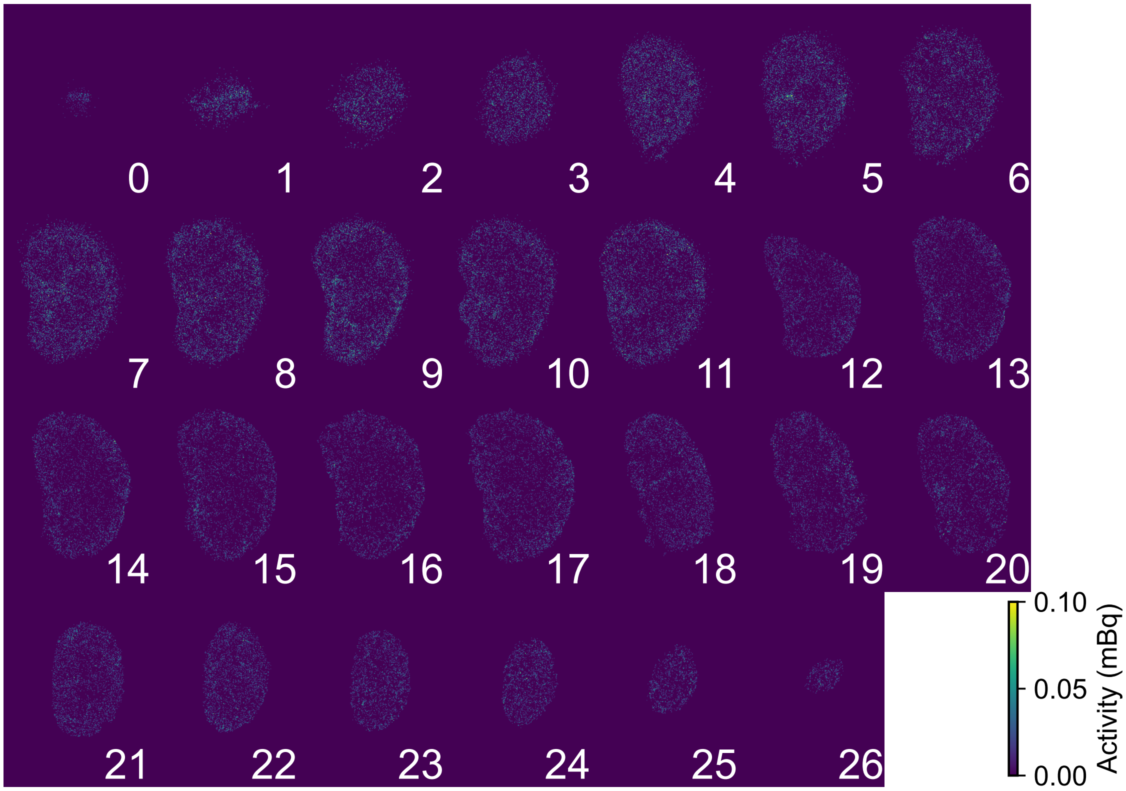

Figure S9: 168 h p.i. right kidney iQID activity DARs for Mouse 1 of 1.

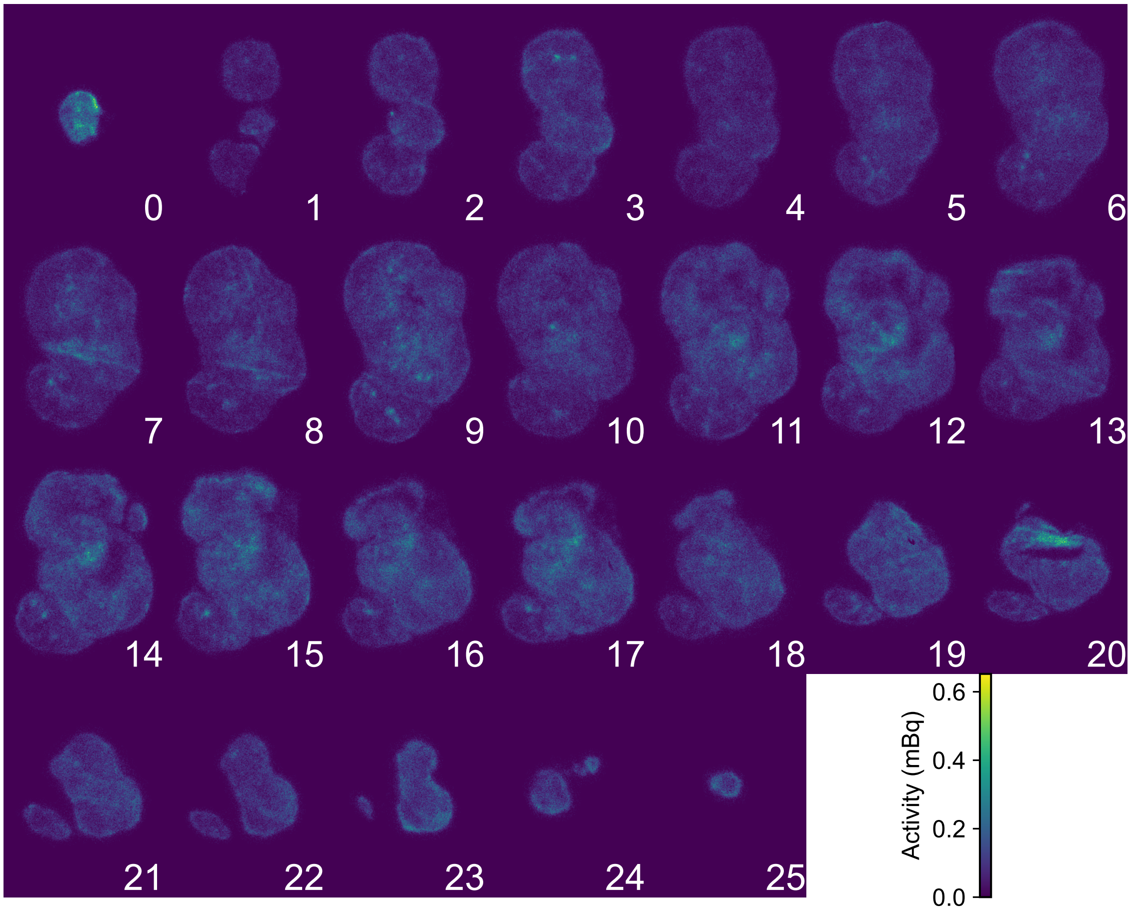

Figure S10: 24 h p.i. tumor iQID activity DARs for Mouse 1 of 2. For analysis, Slice 20 was replaced with a duplicate of an adjacent slice due to damage.

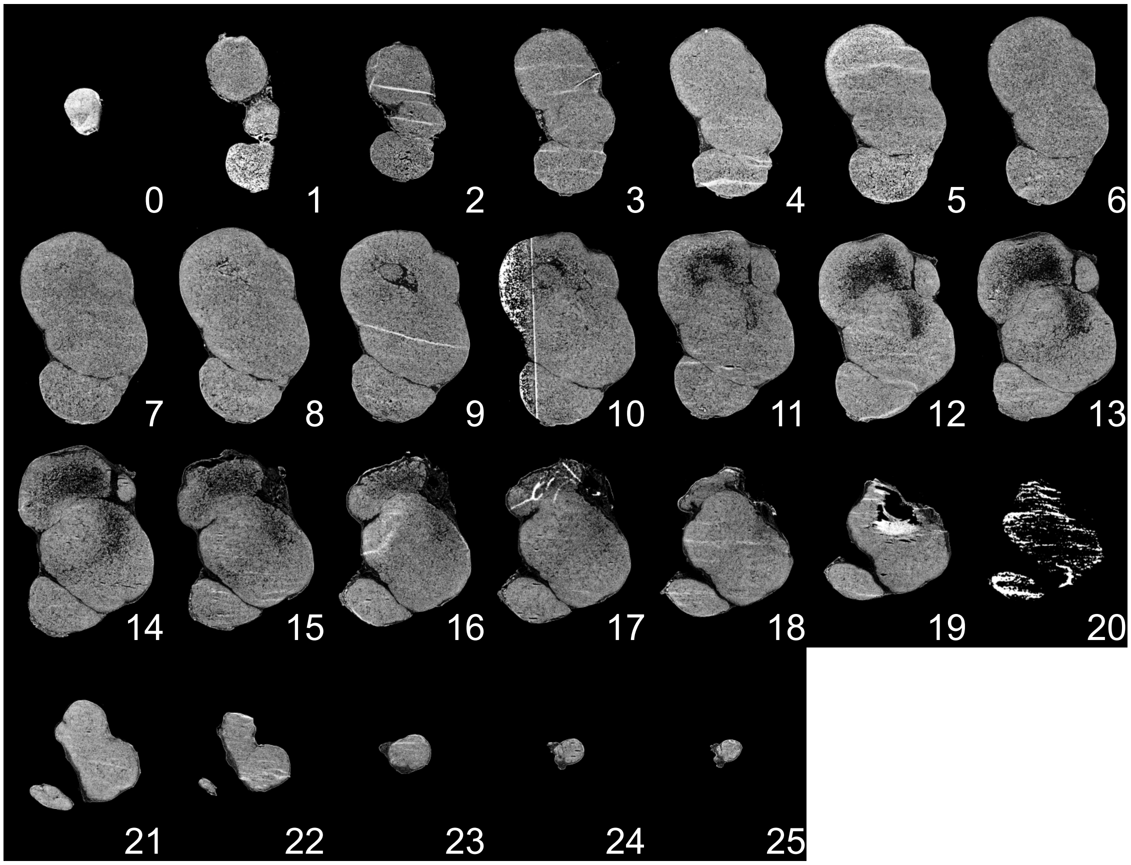

Figure S11: Cell density images from H&E tumor stains for Mouse 1 of 2. Tissue folds in Slice 2, a region of cover glass slippage in Slice 10, and damage in Slices 19-20 are examples of features that were masked out of TCP analysis. Slices where correct registration with the corresponding DAR was not clear were excluded from analysis (e.g. Slice 24).

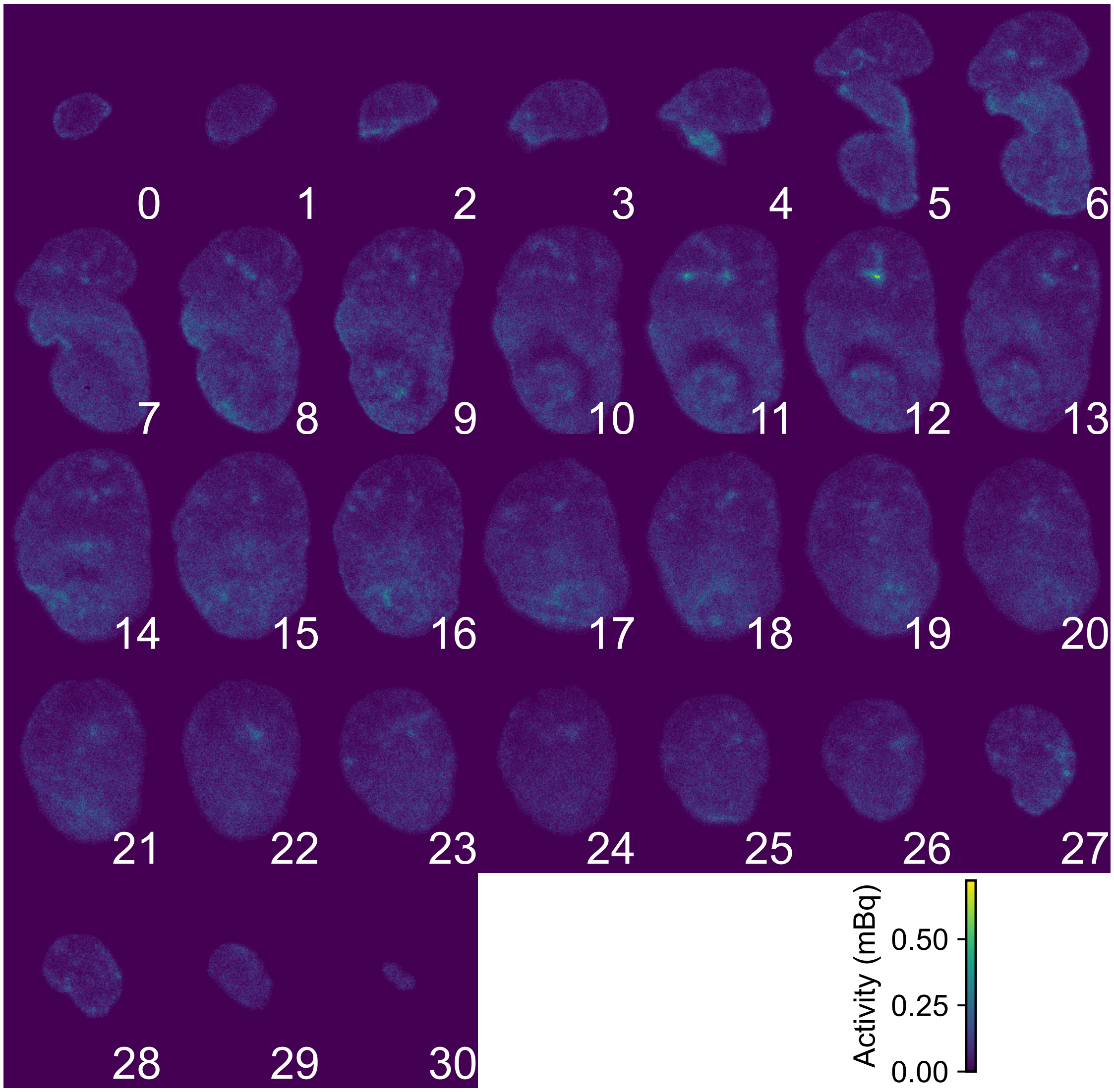

Figure S12: 24 h p.i. tumor iQID activity DARs for Mouse 2 of 2.

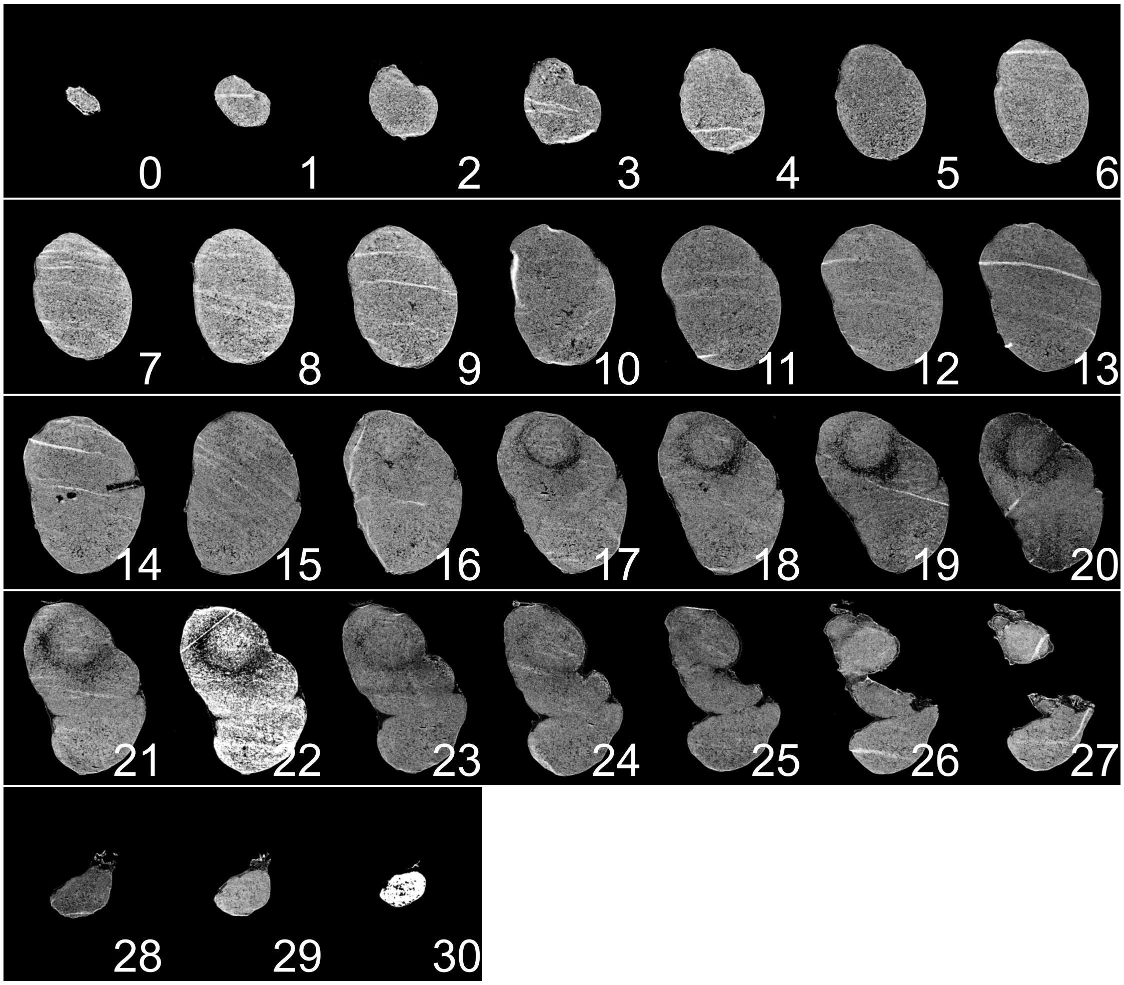

Figure S13: Cell density images from H&E tumor stains for Mouse 2 of 2.

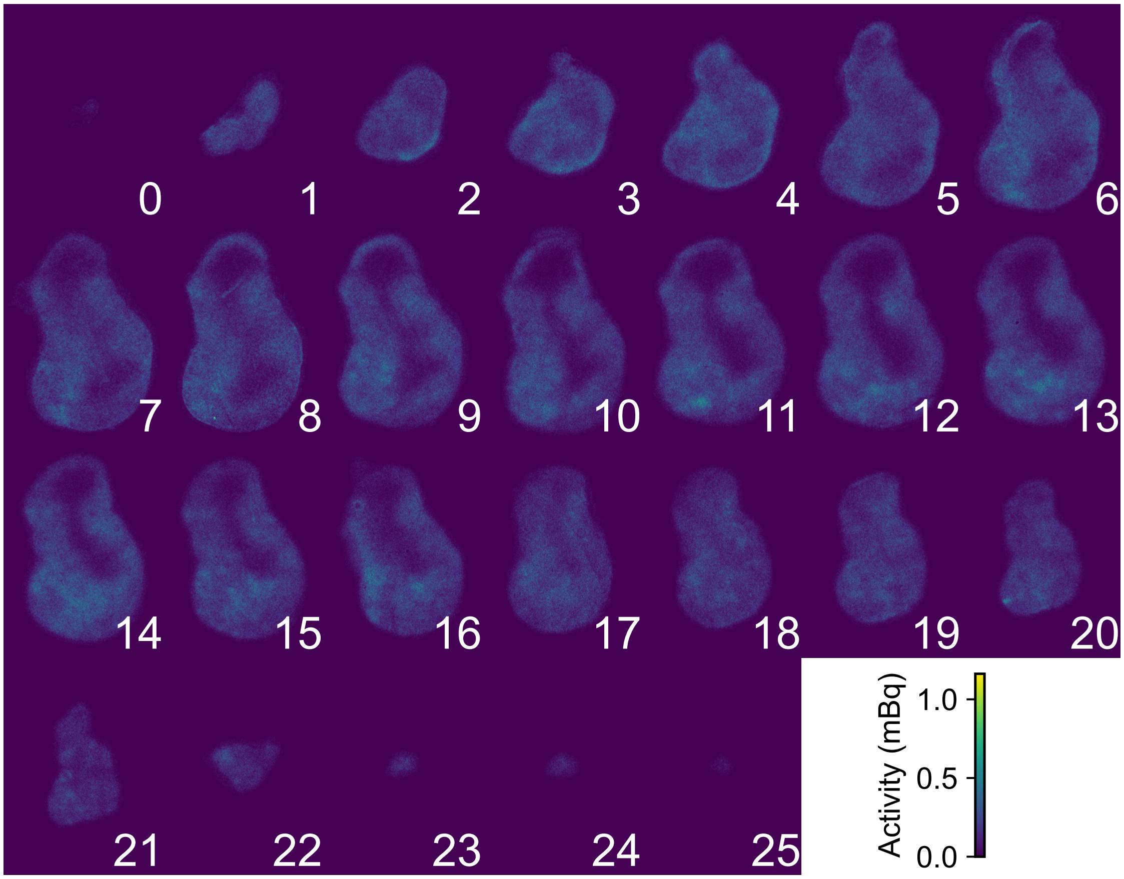

Figure S14: 168 h p.i. tumor iQID activity DARs for Mouse 1 of 2.

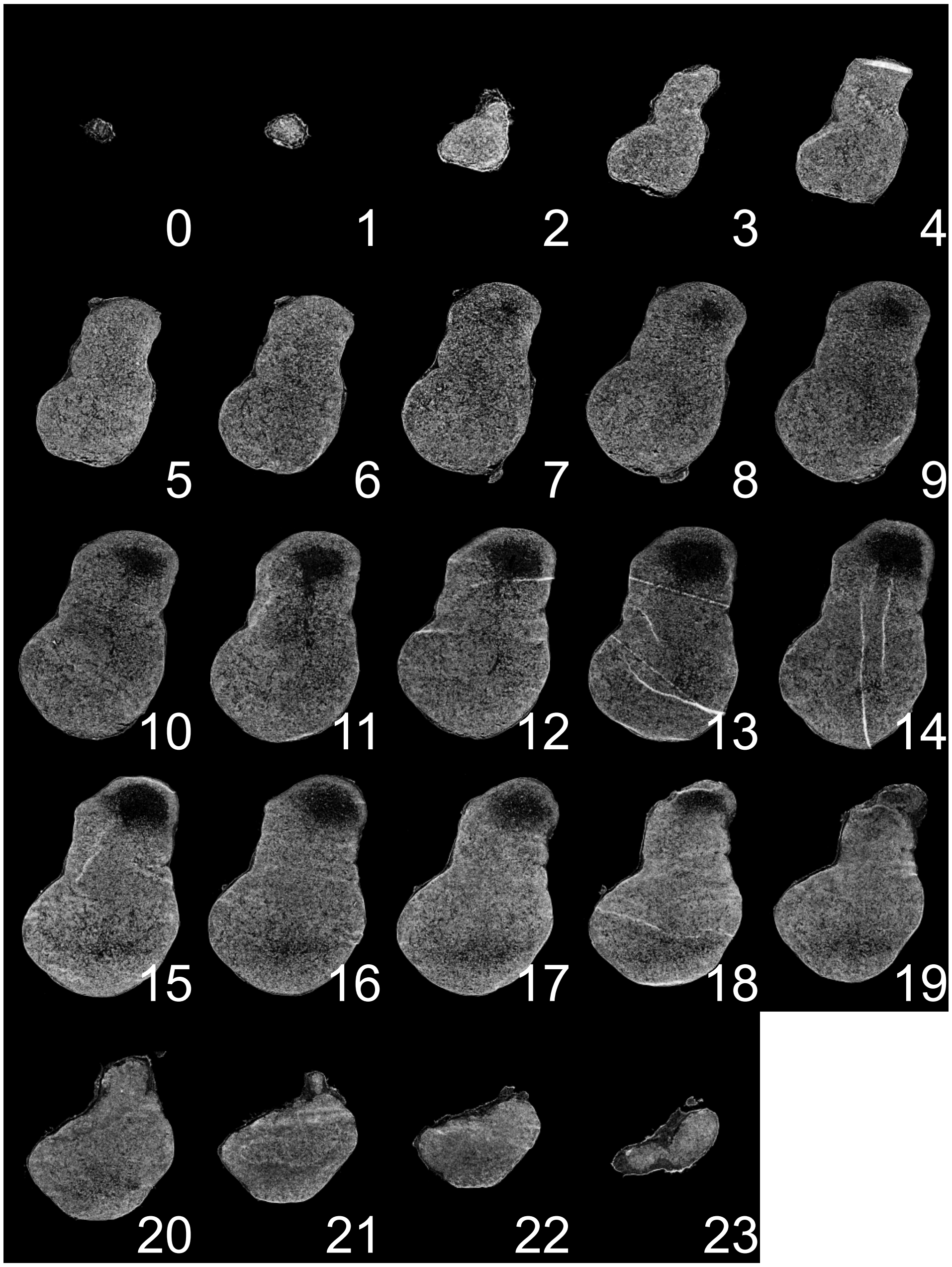

Figure S15: Cell density images from H&E tumor stains for Mouse 1 of 2. Some edge slices were too damaged by the cryotome to create slides.

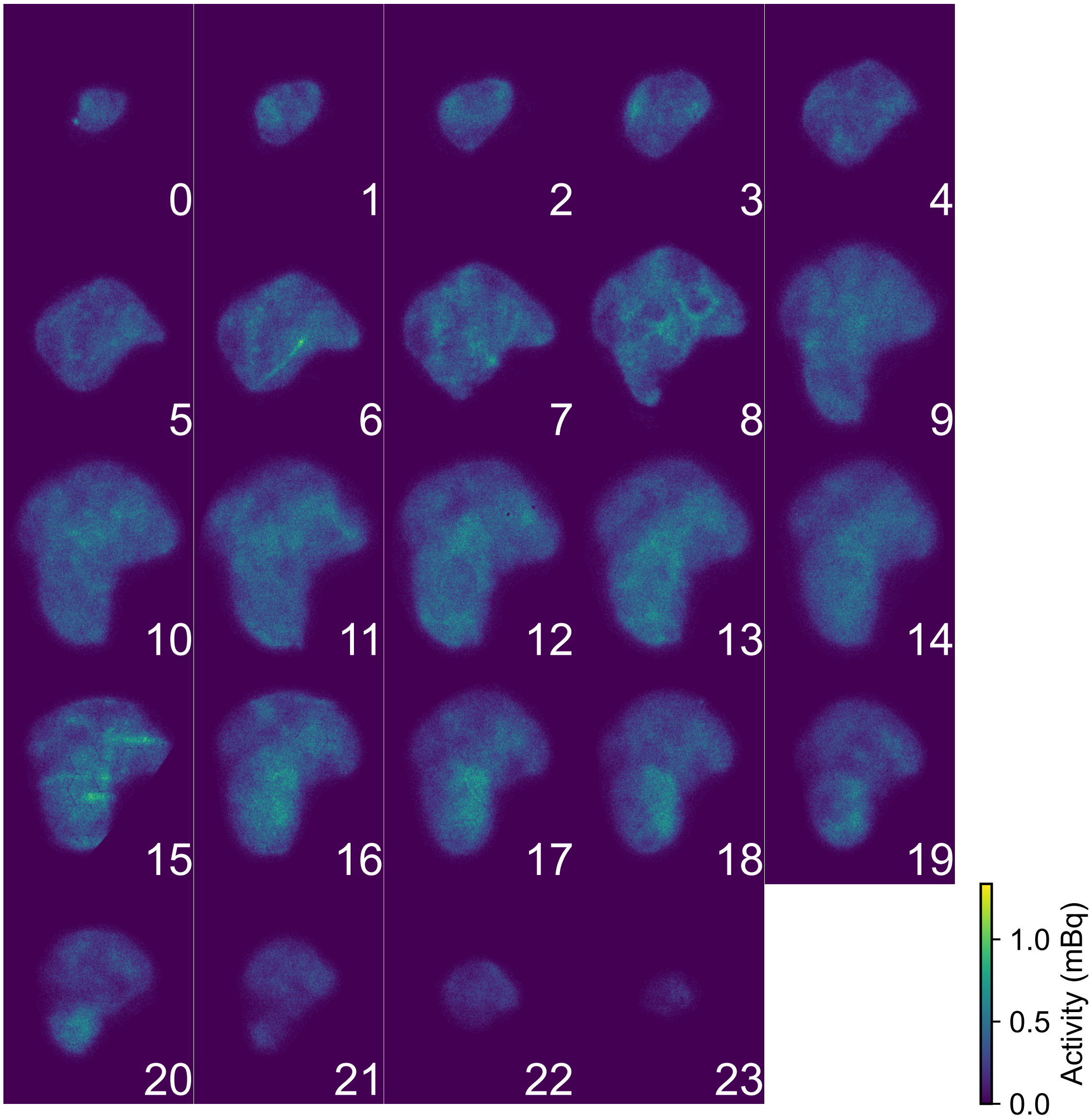

Figure S16: 168 h p.i. tumor iQID activity DARs for Mouse 2 of 2. H&E slides were not obtained for this tumor, and therefore this sample was not processed in the dosimetry procedures described in the main text.

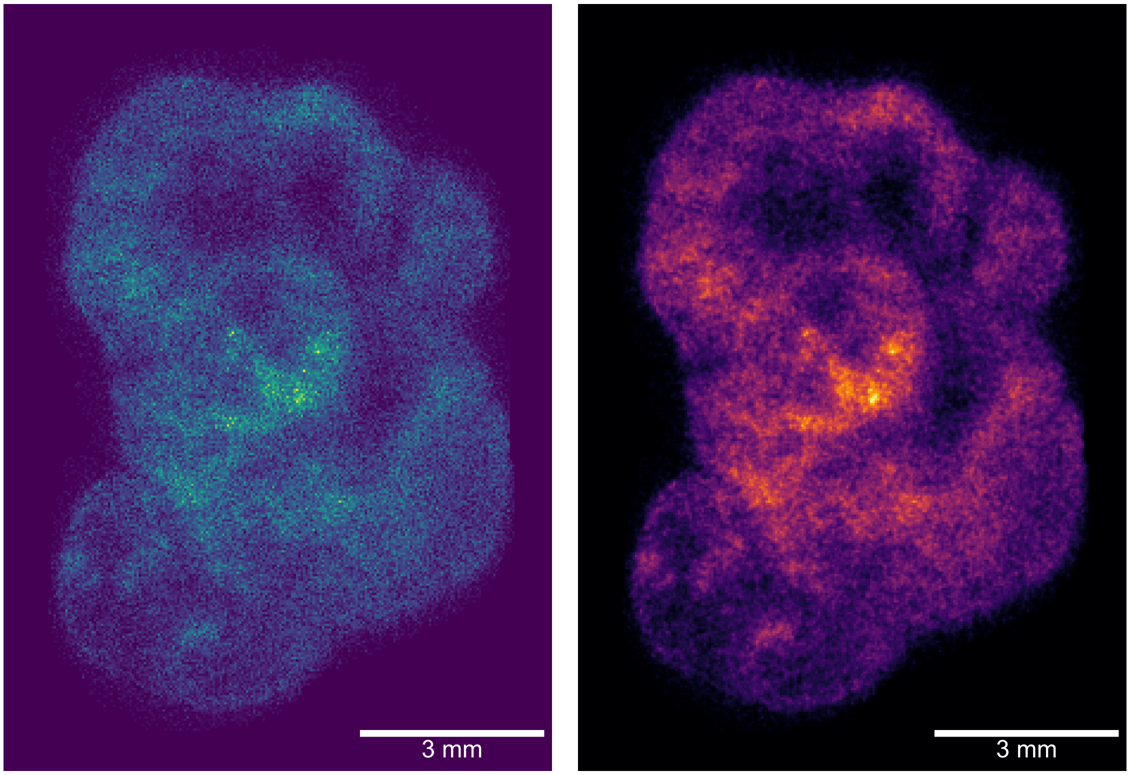

Figure S17: Large-size comparison of example iQID DAR before and after cloning-method DPK convolution (24 h p.i. tumor). Left shows activity image, while right shows dose rate image.

7. References

1. Bobba KN et al. Development of CD46 Targeted Alpha Theranostics in Prostate Cancer Using 134Ce/225Ac-Macropa-PEG4-YS5. Theranostics 2024 Jan; 14:1344–60
2. Krane KS. Introductory Nuclear Physics. John Wiley & Sons, Inc., 1988
3. Castillo Seoane D et al. Gamma Counting Protocols for the Accurate Quantification of 225Ac and 213Bi without the Need for a Secular Equilibrium between Parent and Gamma-Emitting Daughter. EJNMMI Radiopharmacy and Chemistry 2022 Oct; 7:28
